# Characterization of bHLH/HLH genes that are involved in brassinosteroid (BR) signaling in fiber development of cotton (*Gossypium hirsutum*)

**DOI:** 10.1186/s12870-018-1523-y

**Published:** 2018-11-27

**Authors:** Rui Lu, Jiao Zhang, Dong Liu, Ying-Li Wei, Yao Wang, Xue-Bao Li

**Affiliations:** Hubei Key Laboratory of Genetic Regulation and Integrative Biology, School of Life Sciences, Central China Normal University, Wuhan, 430079 China

**Keywords:** Cotton (*Gossypium hirsutum*), bHLH/HLH transcription factor, Fiber development, Phylogenetic analysis, Brassinosteroid (BR) signaling, Gene expression

## Abstract

**Background:**

Basic helix-loop-helix/helix-loop-helix (bHLH/HLH) transcription factors play important roles in plant development. Many reports have suggested that bHLH/HLH proteins participate in brassinosteroid (BR) hormone signaling pathways to promote cell elongation. Cotton fibers are single-cells and derived from seed surface. To explore the roles of bHLH/HLH proteins in cotton fiber development progress by modulating BR signaling pathway, we performed a systematic analysis of the *bHLH/HLH* gene family in upland cotton (*Gossypium hirsutum*) genome.

**Results:**

In this study, we identified 437 *bHLH/HLH* genes in upland cotton (*G. hirsutum*) genome. Phylogenetic analysis revealed that GhbHLH/HLH proteins were split into twenty six clades in the tree. These *GhbHLH/HLH* genes are distributed unevenly in different chromosomes of cotton genome. Segmental duplication is the predominant gene duplication event and the major contributor for amplification of *GhbHLH/HLH* gene family. The *GhbHLH/HLHs* within the same group have conserved exon/intron pattern and their encoding proteins show conserved motif composition. Based on transcriptome data, we identified 77 *GhbHLH/HLH* candidates that are expressed at relatively high levels in cotton fibers. As adding exogenous BR (brassinolide, BL) or brassinazole (Brz, a BR biosynthesis inhibitor), expressions of these *GhbHLH/HLH* genes were up-regulated or down-regulated in cotton fibers. Furthermore, overexpression of GhbHLH282 (one of the BR-response genes) in Arabidopsis not only promoted the plant growth, but also changed plant response to BR signaling.

**Conclusion:**

Collectively, these data suggested that these *GhbHLH/HLH* genes may participate in BR signaling transduction during cotton fiber development. Thus, our results may provide a valuable reference data as the basis for further studying the roles of these *bHLH/HLH* genes in cotton fiber development.

**Electronic supplementary material:**

The online version of this article (10.1186/s12870-018-1523-y) contains supplementary material, which is available to authorized users.

## Background

Cotton, one of the most important economic crops, supplies the largest number of natural fibers in the textile market around the world. The cotton genus (*Gossypium*) is composed of nearly 50 species, of which four species, including two diploids (*G. arboreum* and *G. herbaceum*, 2n = 2× = 26 AA) and two allotetraploids (*G. hirsutum* and *G. barbadense*, 2n = 4× = 52 AADD), have been widely cultivated for the commercial values of fibers [1]. Upland cotton (*G. hirsutum*) accounts for the largest planting area among the varieties of cotton crops and provides most of the valuable fibers needed by modern textile industry. Fiber development is divided into four overlapping stages, fiber initiation, elongation, secondary cell wall thickening and maturity [2]. The cotton fiber is regarded as a powerful cell research model since it is an easily isolated single cell with distinct stages of cotton fiber cell development. In vitro ovules culture can be carried out to make further research about detailed development stages of cotton fibers [3, 4].

Basic helix loop helix/helix loop helix (bHLH/HLH) proteins were named for the basic helix loop helix domains. The bHLH domains are comprised of 50–60 amino acids with two functionally different regions, the N terminal basic region and the C terminal helix-loop-helix region. The basic region, consisting of around 15 amino acids with numerous basic residues, can recognize and bind to DNA [5, 6]. The HLH region, including two amphipathic α-helices that are parted by a loop region with alterable sequence, can form the dimers with other HLH domains [7]. In higher plants, the bHLH/HLH proteins participate in regulation of plant growth and development, such as light signal transmission, plant hormone signals and organs development. For example, in light signal transmission, AtPIF4 takes part in regulating photomorphogenesis mainly by interacting with photochromes [8]. AtSPT is not only related to Arabidopsis sterility, but also has a coordinating relationship with DELLA protein to regulate the expression of gibberellins (GA) response genes jointly. *AtSPT* mutation can remove its inhibition of cell growth [9, 10]. AtGL3 is also involved in the development of Arabidopsis trichomes by contributing to form the trichome promoting comlpex TTG1/GL3 (EGL3) /GL1 [11, 12]. In the stem or leaf epidermal cells of Arabidopsis, the complex can induce the expression of the downstream gene *GL2* to determine the development of epidermal cells. And the MYB proteins, AtCPC, AtTRY, AtTCL1 and AtETC1, can bind to GL3 competing with GL1, thereby negatively regulating the initiation of trichomes [13, 14].

Brassinosteroid (BR) signalling is a well-described signalling pathway in Arabidopsis that plays a crucial role in plant growth and development. Many bHLH/HLH protiens have been reported to be involved in the BR signaling transduction. AtbHLH064 (HBI1), AtbHLH158 (IBH1), AtbHLH044/058/050 (BEE1/2/3), AtbHLH046/102/141 (BIM1/2/3), AtbHLH136/135 (PRE1/3) and AtbHLH150/148/147/149 (AIF1/2/3/4) are regulated by BZR1/BES1 to influence BR signaling in Arabidopsis [15–19]. Most of them can form homodimers and heterodimers, endowing them with the capacity to function in the regulation of multiple transcriptional programs [20]. For example, AtCESTA can form heterodimer with AtbHLH044 (BEE1) to regulate the expression of BR biosynthesis related gene *CPD* positively [21]. AtbHLH064 (HBI1) promotes cell elongation by regulating downstream genes expression and its functions can be inhibited by AtbHLH158 (IBH1), which inhibition can be relieved by the interaction of AtbHLH136 (PRE1) and AtbHLH158 (IBH1) [22, 23]. A report also demonstrates that AtbHLH136 (PRE1), AtbHLH158 (IBH1) and AtbHLH049/074/077 (ACE1/2/3) constitute a triantagonistic bHLH system that competitively regulates cell elongation [24, 25].

Exogenous application of BL (BR) promotes cotton fiber cell elongation while treatment of cotton floral buds with Brz (a BR inhibitor) results in the complete absence of fiber cell differentiation, indicating that BR is required for fiber initiation and elongation [26, 27]. Besides, our previous study indicated that Gh14–3-3 proteins are involved in regulating fiber initiation and elongation through their interacting with GhBZR1 to modulate BR signalling [28]. Also, cotton bHLH/HLH transcription factors may play important roles in fiber development [29–31]. However, little is known about how bHLH/HLH transcription factors modulate BR signaling during fiber development of cotton in detail so far. In our study, we genome-widely identified the cotton *bHLH/HLH* genes that may be involved in BR signaling in fiber development. Furthermore, the characters of these cotton bHLH/HLH transcription factors were approached in detail.

## Results

### Characterization of cotton bHLH/HLH transcription factors

To identify the *bHLH/HLH* transcription factor genes in upland cotton (*G. hirsutum*) genome, all published *bHLH/HLH* gene sequences of *Arabidopsis* and rice were employed as queries to perform homologous blast searches against the cotton genome database (https://www.cottongen.org/tools/blast/blast) [32]. Originally, 498 candidate bHLH/HLH genes were identified in cotton. Among them, 61 repeated sequences were discarded. Furthermore, to evaluate the reliability of the initial results, the conserved bHLH/HLH domain of predicted bHLH/HLH proteins were confirmed using the Hmmscan program and the pfam tools (http://www.ebi.ac.uk/Tools/hmmer/search/hmmscan) according to reported methods [33]. The results showed that all the 437 putative genes have conserved bHLH or HLH domains in their sequences. Since there was no uniform annotation for the *GhbHLH/HLH* genes, the *bHLH/HLH* genes are named as *GhbHLH001* to *GhbHLH437* according to their chromosome locations. The average length of the newly identified bHLH/HLH proteins is 353 amino acids with variation range of 73 to 1302. The characters of the bHLH/HLH proteins including the amino acid numbers, molecular weights (MW), theoretical isoelectric points (pI) and chromosome locations were listed in Additional file 1: Table S1.

### Phylogenetic relationship of cotton bHLH/HLH proteins

**Fig. 1:**
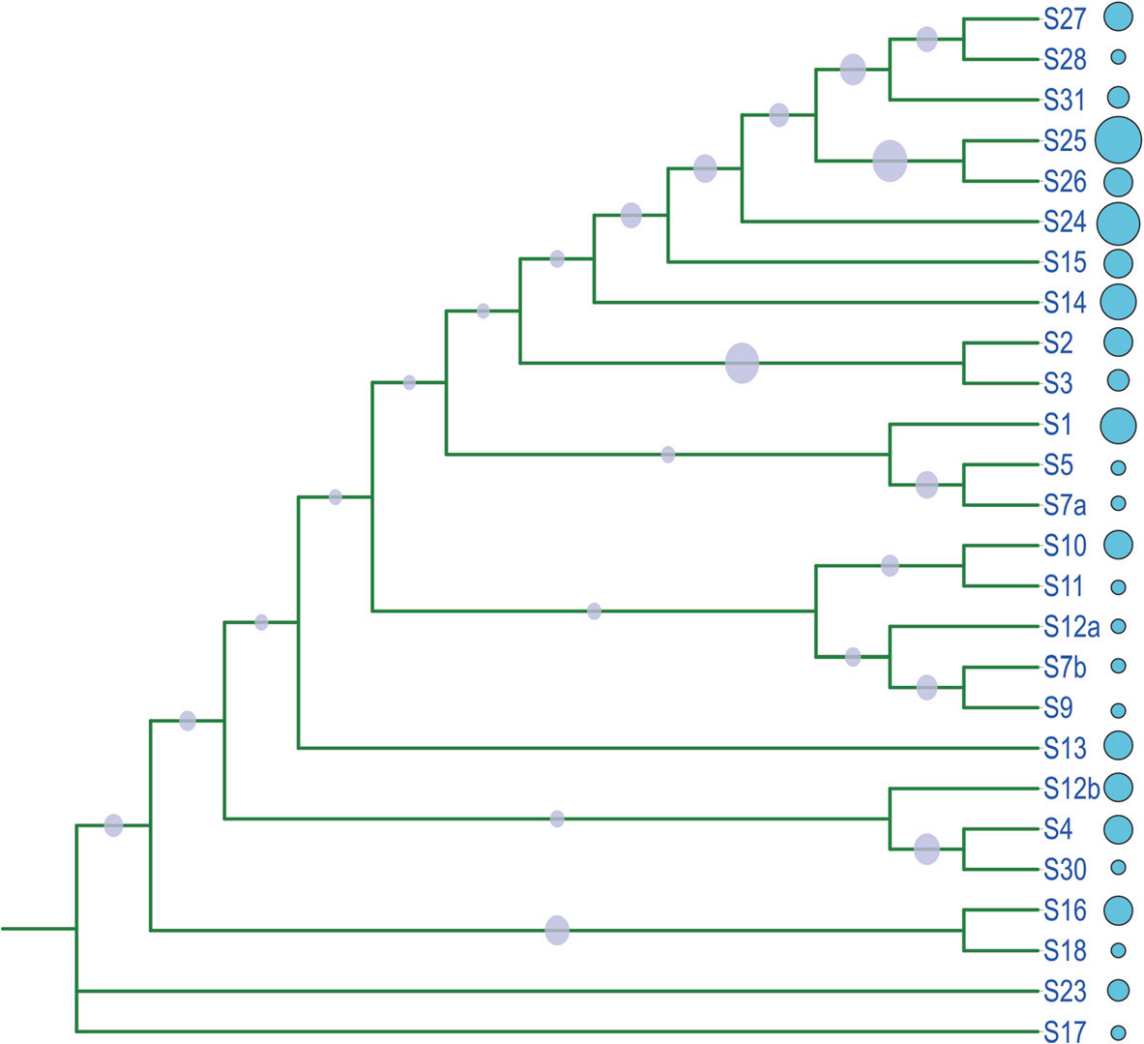
Phylogenetic relationship of cotton bHLH/HLH transcription factors*.* MEGA 6.0 software was employed to construct an unrooted phylogenetic tree based on alignments of 437 bHLH/HLH domains identified from upland cotton (*G. hirsutum*) using the NeighborJoining (NJ) method with the following parameters: The number of differences model, pairwise deletion and 1000 bootstraps. Subfamilies are collapsed and represented as grey dots with area proportional to member numbers of each subfamily

To exmaine the evolutionary history and phylogenetic relationship of upland cotton bHLH/HLH proteins, an unrooted phylogenetic tree was constructed using the Neighbor Joining (NJ) method based on the results of multiple sequence alignment of the identified 437 GhbHLH/HLH domain sequences with 172 Arabidopsis bHLH/HLH domain sequences and one rice bHLH domain sequence (Additional file 1: Figure S1). To verify the reliability of the constructed phylogenetic tree, the Maximum likelihood, Minimal Evolution and PhyML methods were employed to reconstruct the phylogenetic trees of bHLH/HLH transcription factors respectively. The phylogenetic trees constructed by the four methods were identical with only slight differences in some branches. Another unrooted phylogenetic tree was also constructed using the Neighbor Joining (NJ) method based on the results of multiple sequence alignment of the 437 GhbHLH/HLH domain sequences (Fig. 1). Subsequently, the analyses of gene structures, motif sites distribution and gene expression patterns also confirmed the validity of the phylogenetic relationship of GhbHLH/HLH protein sequences.

On the basis of the NJ phylogenetic tree, the 610 bHLH/HLH protein sequences were grouped into 26 subfamilies (including S1–S5, S7a, S7b, S9–S18 and S23–S31) (Additional file 1: Figure S1), which remain in the classification of bHLH/HLH transcription factors by Lorenzo Carretero-Paulet [34]. Additionally, S7 and S12 were split into two subgroups (S7a and S7b, S12a and S12b), while S18, S19, S20, S21 and S22 were clustered into a single subfamily in the tree. S25, the largest group among the whole gene family groups, is composed of 61 bHLH/HLH members, occupying 14% of the total bHLH/HLH numbers, but S18 contain 2 members, being the smallest clade in the tree (Additional file 1: Table S2). The interspersed distribution of the bHLH/HLH proteins in most clades of the tree implied that the bHLH/HLH proteins expanded before the divergence of the lineages. Most of Arabidopsis *bHLH/HLH* genes have two or more correspondences in upland cotton genome, indicating that the divergence of cotton and Arabidopsis occurred before *GhbHLH/HLH* genes duplication. In subfamily 8, neither *GhbHLH/HLH* nor *AtbHLH/HLH* sequences was classified into rice *bHLH/HLH* sequences, which implied that this group may be lost after the divergence of monocots and dicots.

### Chromosomal distribution and duplication of cotton *bHLH/HLH* genes

**Fig. 2:**
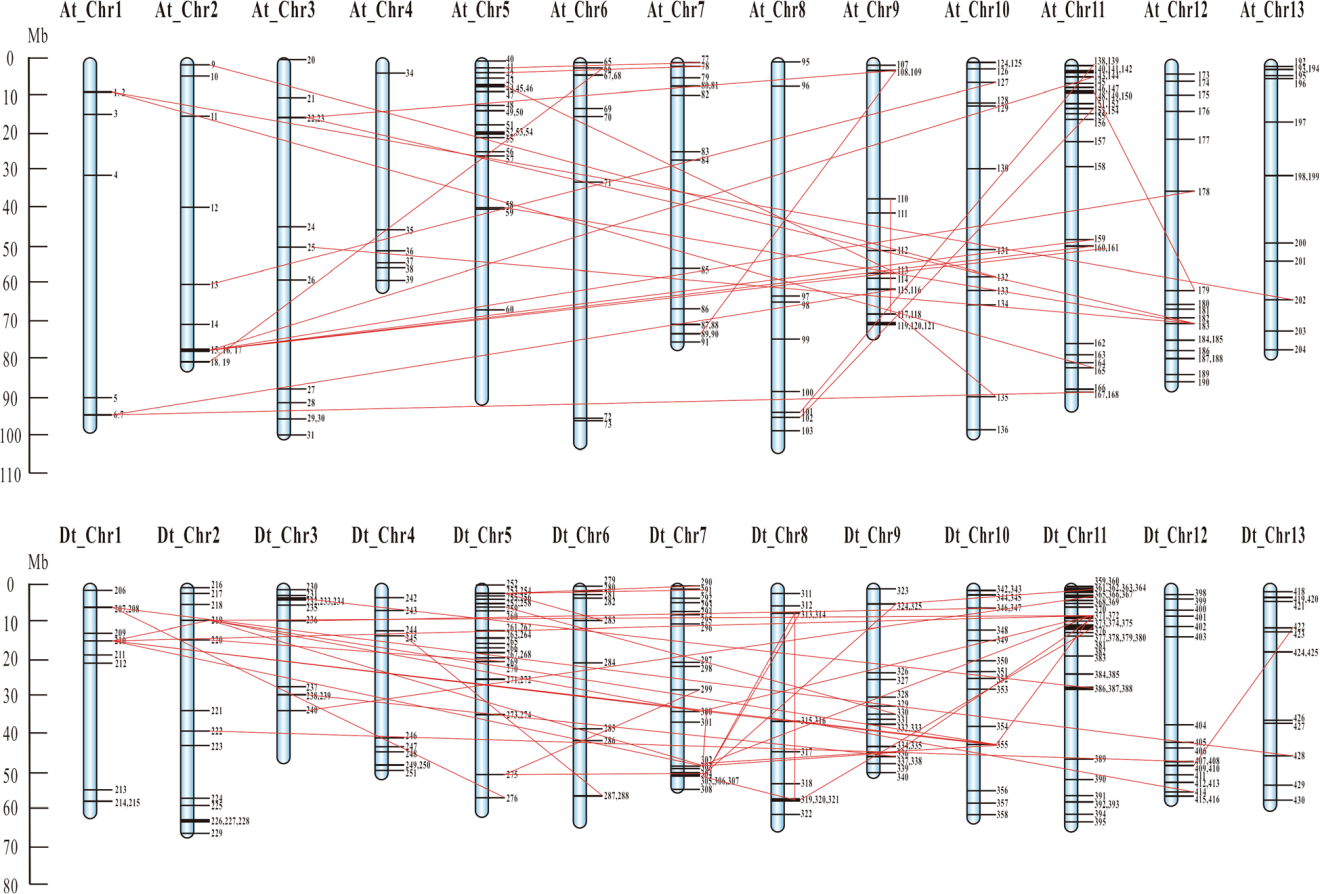
Chromosomal distribution and duplication of cotton (*G. hirsutum*) *bHLH/HLH* genes. The scale is in megabases (Mb). The chromosome number is indicated at the top of each chromosome. The paralogous bHLH genes are linked with a red line

To clarify the chromosomal distribution of the *GhbHLH/HLH* genes, the physical locations of all the *GhbHLH/HLH* genes were acquired through the blastn searches against upland cotton genome databases. Among the 437 genes, a total of 397 genes are unevenly distributed on different chromosomes of upland cotton genome, while the remaining 40 genes were unmapped on scaffolds. Upland cotton (*G. hirsutum*) genome includes A-subgenome and D-subgenome, containing 205 *bHLH/HLH* and *224 bHLH/HLH* genes, respectively. In our study, the genes distribution event on A-genomes is similar to that in D-genome. For example, Chromosome A11 of A-subgenome has the largest number of *bHLH/HLH* genes, in accordance with the fact that Chromosome D11 of D-subgenome has the largest number of *bHLH/HLH* genes. At the same time, the fewest number of *bHLH/HLH* genes were found on A1 and D1 chromosomes (Fig. 2).

To clear the clouds on the mechanism behind the expansion of *GhbHLH/HLH* gene family, gene duplication events about the evolution of upland cotton genome were analyzed. 19 gene pairs in A-subgenome and 21 pairs in D-subgenome were identified respectively. These gene pairs were gathered in the same clade of the phylogenetic tree with high similarity. For example, the sequence of *GhbHLH013* shares 94.5% sequence similarity with the sequence of *GhbHLH127*. Among these paralogous gene pairs, 18 pairs in A-subgenome and 19 pairs in D-subgenome are located on different chromosomes, indicating that segmental duplication event plays the leading role in *bHLH/HLH* gene family amplification during evolution, and no tandem duplication event was observed in these identified gene pairs (Fig. 2).

### Structures and conserved motifs of cotton *bHLH/HLH* genes

**Fig. 3:**
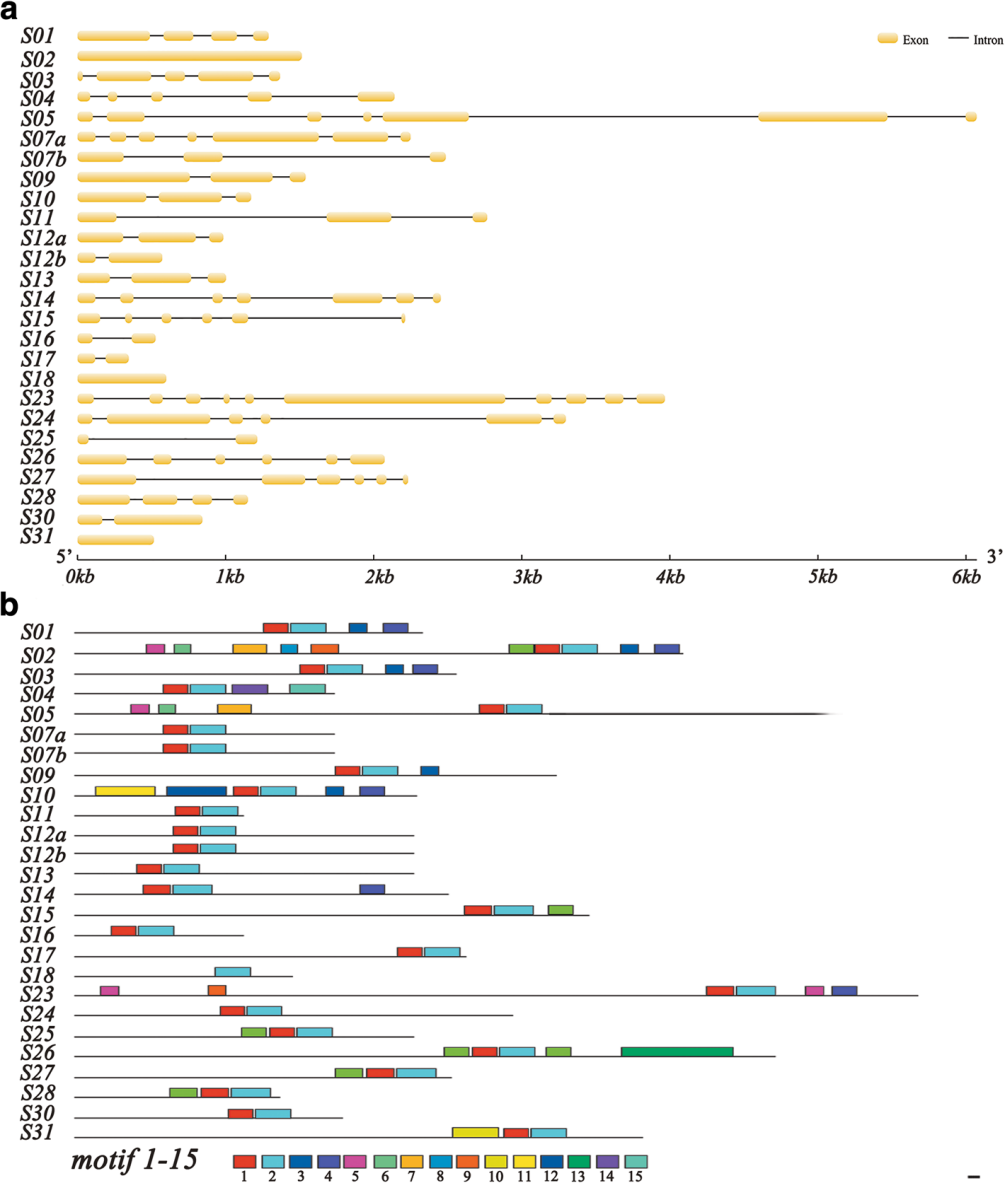
Characterization of cotton (*G. hirsutum*) *bHLH/HLH* genes and their encoding proteins*.* **a** The conserved distribution of exons and introns in *GhbHLH/HLH* genes. The yellow columns represent exons and black lines indicate introns. **b** Conserved motifs of GhbHLH/HLH proteins. The motifs 1–15 were identified using MEME program, and each motif is shown with a specific color. The composition elements of all the 15 motifs are shown in Additional file 1: Figure S2

For further insights into the evolutionary relationships of *GhbHLH/HLH* genes, we observed the exon/intron structures of *GhbHLH/HLH* genes by alignment of the genomic DNA sequences of these genes with their corresponding cDNA sequences. Another unrooted phylogenetic tree was constructed to determine if the exon/intron pattern is consistent with the phylogenetic classification. As expected, most *GhbHLH/HLH* genes in the same group show similar exon/intron pattern in terms of exon length and intron number (Fig. 3a).

To explore the motif composition in GhbHLH/HLH proteins, conserved motif sites were searched by online program MEME. Fifteen conserved motifs (named motif 1–15) were identified in the GhbHLH/HLH protein sequences (Additional file 1: Figure S2). Most of the GhbHLH/HLH proteins within the same subfamily share similar motifs, while high divergence occurs among different subfamilies, indicating that the same subfamily members may perform similar roles in cotton. For instance, the bHLH/HLHs in subfamily 1 share the conserved motif 1, 2, 3 and 4, while the members in subfamily 5 contain the conserved motifs 1, 2, 5, 6 and 7. Besides, some subfamilies have specific motifs particularly (e.g. motif 15 for subfamily 4, motif 11 for subfamily 10, and motif 13 for subfamily 26), suggesting that these motifs may lead to the specific functions of the individual subfamily. Additionally, Prosite online program was employed to annotate the functions of the identified 15 motifs. The results showed that only motif 1 and motif 2 had hits for PROSITE motifs in the database. Both of the motifs were annotated as conserved HLH domain and were uniformly observed in all GhbHLH/HLH proteins (Fig. 3b, Additional file 1: Figure S2). Collectively, the results revealed that the exon/intron patterns of *GhbHLH/HLH* genes and the motif compositions of GhbHLH/HLH proteins are consistent with the phylogenetic classification of GhbHLH/HLHs in cotton.

### Expression of *bHLH/HLH* genes in fibers is induced by Brassinosteroid (BR)

**Fig. 4:**
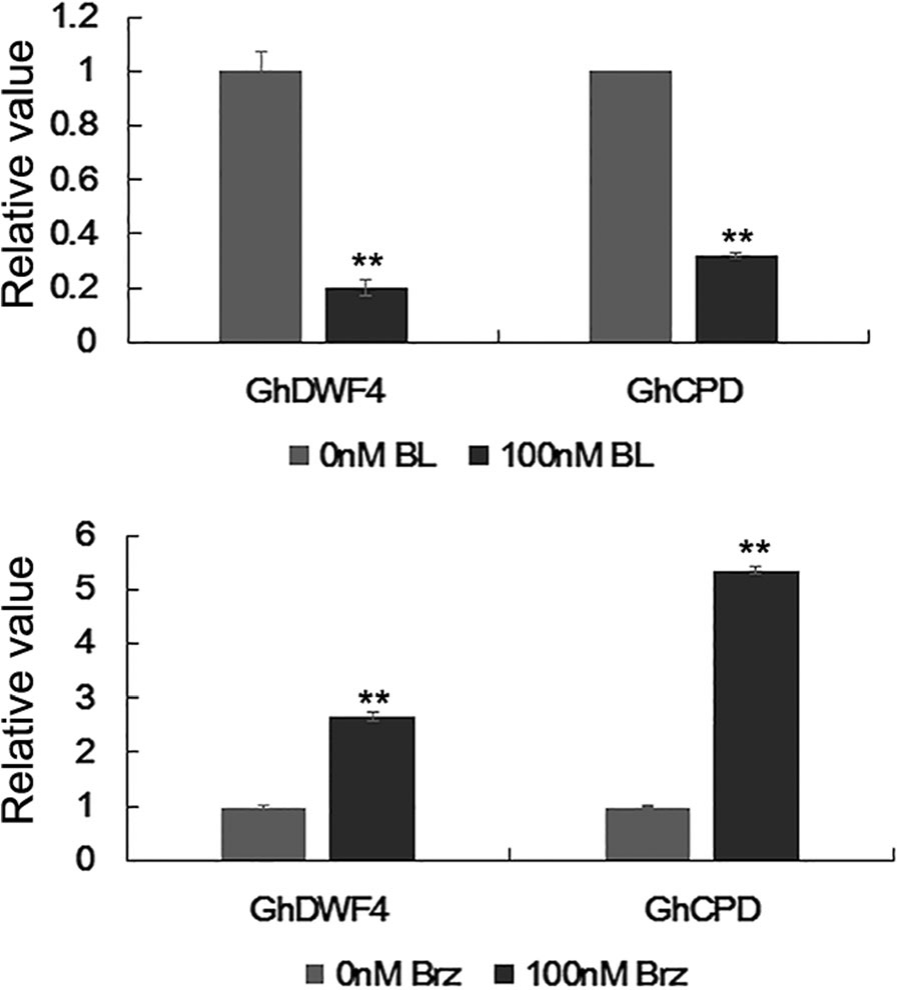
Quantitative RT-PCR analysis of expression of *GhCPD* and *GhDWF4* genes in cotton fibers treated with BL and Brz. Cotton bolls (9 DPA) were in vitro cultured in liquid BT medium without or with 100 nM 2, 4-epibrassinolide (eBL) or brassinazole2001 (Brz) at 30 °C in darkness for 3 h. Transcript levels of *GhCPD* and *GhDWF4* were determined by quantitative RT-PCR, using *GhUBI1* (EU604080) as a quantification control, and expression levels of the genes in the untreated samples (0 nM BL or Brz) were set to 1. Data were processed with Microsoft Excel. Mean values and standard deviations are shown from three independent experiments. Two asterisks represent there was very significant difference (*P* < 0.01) in gene expression level between the treated sample and the untreated sample (control). DPA, day post anthesis

**Fig. 5:**
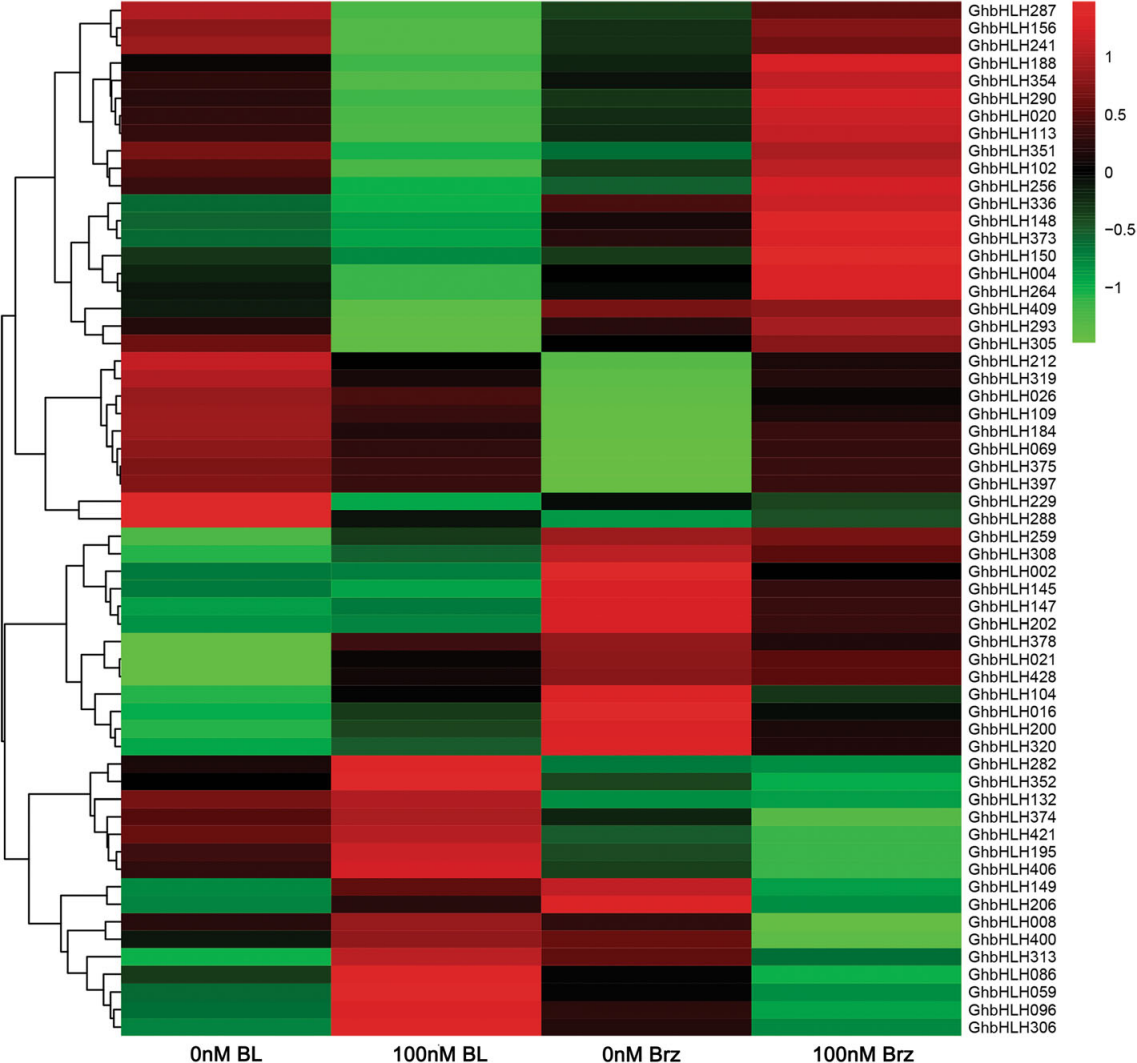
Heatmap representation for expression profiles of cotton (*G. hirsutum*) *bHLH/HLH* genes in 9 DPA fibers under BL and Brz treatments. Transcript levels of *GhbHLH/HLH* genes were determined by quantitative RT-PCR, using *GhUBI1* as a quantification control. Data were processed with Microsoft Excel. The values were normalized and the average of three biological replicates was used to generate log2 expression value. Then log2-transformed values were subjected to R sofware (15.2) for expression analysis. Heatmaps for gene expression patterns were generated by the online program Omicsharea (http://www.omicshare.com/tools/Home/Soft/heatmap). DPA, day post anthesis

To find out the candidate *bHLH/HLH* genes involved in cotton fiber development, the public data of expression profiles of cotton genes in different organs/tissues, including ovules (− 3, 0 and 3 DPA), fibers (5, 10, 20 and 25 DPA), torus, stems, stamens, roots, leaves, pistils, petals and anthers were searched and analyzed for identifying the fiber preferential *bHLH/HLH* genes. 77 *bHLH/HLH* candidates were identified as cotton fiber preferentially expressed genes (Additional file 1: Figure S3). Subsequently, expressions of these genes were analyzed in developing fibers with 2,4-epibrassinolide (BL) and brassinazole2001 (Brz, a BR inhibitor) treatments. It has been reported that *GhCPD* and *GhDWF4*, two BR biosynthesis genes, are feedback-inhibited by BR, and expressions of these genes are significantly suppressed by exogenous BL and promoted by Brz [35, 36]. To test if the above experiments are effective, we firstly analyzed the expression levels of *GhCPD* and *GhDWF4* in fibers with BL and Brz treatments. Our results showed that the transcripts of *GhCPD* and *GhDWF4* were decreased observably by five folds in 3-h cultured ovules with the treatment of 100 nM BL relative to the controls, while increased dramatically by three folds in 3-h cultured ovules with the treatment of 100 nM Brz compared with the controls (Fig. 4). Subsequently, the expression levels of 77 *bHLH/HLH* candidates were analyzed in cotton fibers with treatments of 100 nM BL and 100 nM Brz, respectively. As shown in Fig. 5, expression levels of 59 *bHLH/HLH* genes were altered in fibers under the treatment of BL or Brz. Among these candidates, 27 *bHLH/HLH* genes showed their moderately increased expression levels in fibers with BL treatment as well as moderately decreased expression levels in fibers with Brz treatment. Strikingly, expressions of seven genes (*GhbHLH059/086/096/149/206/306/313*) were largely up-regulated in fibers treated with 100 nM BL, and down-regulated in fibers with the treatment of 100 nM Brz, suggesting that these BL-induced *bHLH/HLH* genes may act as regulators in cotton fiber development by responding to BR signaling. The other *bHLH/HLH* genes showed the decreased expression levels in fibers with BL treatment and the increased expression levels in fibers with Brz treatment. Thus, the results suggested that a number of *GhbHLH/HLH* genes may be involved in response to BR signaling for regulating fiber development of cotton.

### The *bHLH/HLH* genes are differentially expressed during fiber developmental stages

**Fig. 6:**
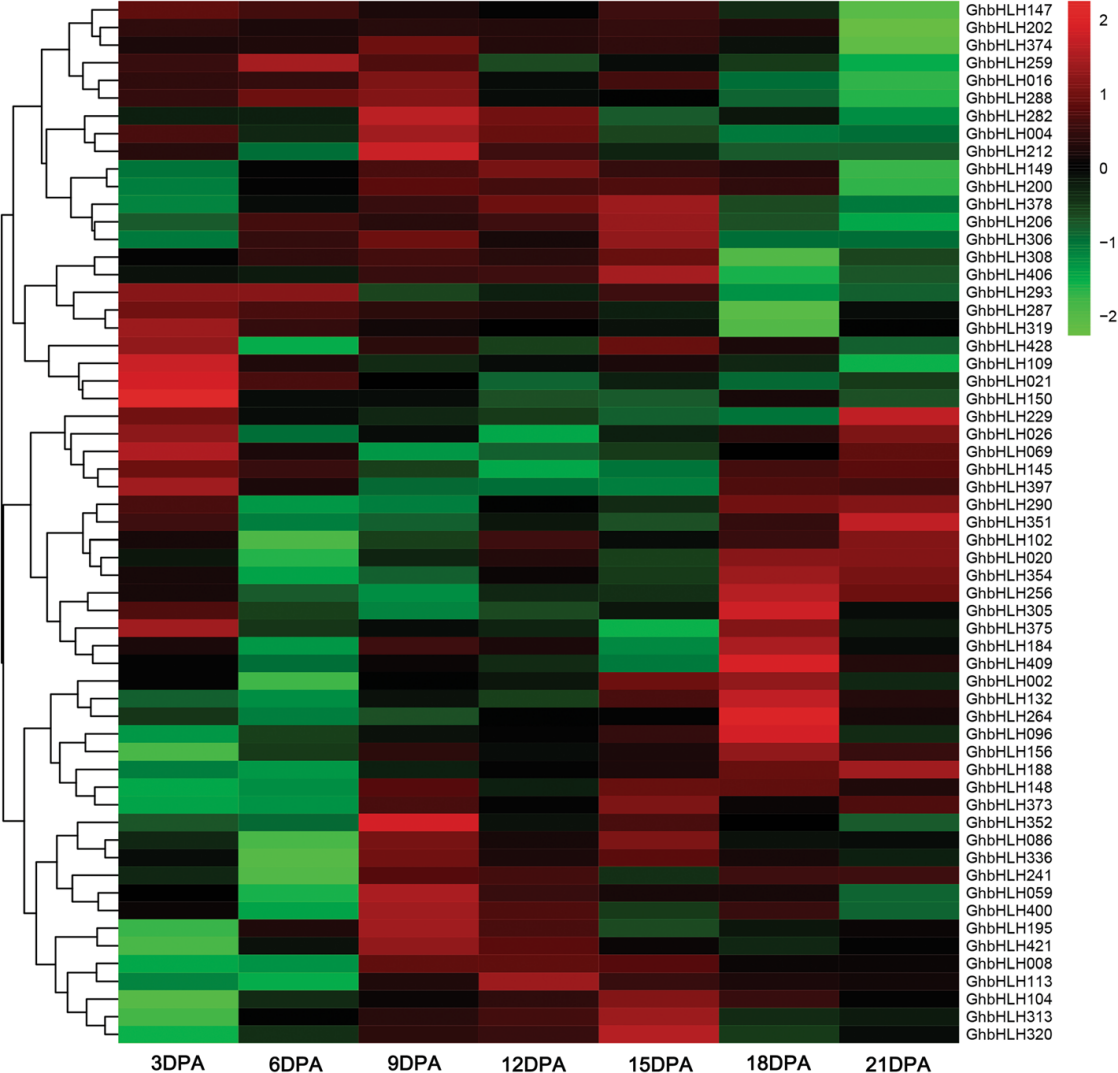
Heatmap representation for expression profiles of cotton (*G. hirsutum*) *bHLH/HLH* genes in cotton fibers. Transcript levels of the *bHLH/HLH* genes were determined by quantitative RT-PCR, using *GhUBI1* as a quantification control. Data were processed with Microsoft Excel. The values were normalized and the average of three biological replicates was used to generate log2 expression value. Then log2-transformed values were subjected to R sofware (15.2) for expression analysis. Heatmaps for gene expression patterns were generated by the online program Omicsharea (http://www.omicshare.com/tools/Home/Soft/heatmap). 3DPA – 21DPA, 3 to 21 DPA (day post anthesis) fibers of cotton

To further clarify the expression profiles of the BR-responsive *bHLH/HLH* genes in cotton fibers, quantitative RT-PCR (qRT-PCR) was performed to detect expression levels of the identified candidate *bHLH/HLH* genes in fibers at different developmental stages (3, 6, 9, 12, 15, 18, and 21 DPA). As shown in Fig. 6, most of the identified *bHLH/HLH* genes displayed relatively higher expression levels from 3 to 15 DPA fibers, while the transcripts of a few genes were accumulated in 18 to 21 DPA fibers. For example, *GhbHLH021/109/150/319/428* were expressed at relatively high expression levels in 3 DPA fibers, suggesting that they may regulate very early fiber elongation, and perhaps fiber initiation, of cotton. On the other hand, the transcripts of *GhbHLH004/059/086/148/195/241/282/336/352/373/400/421* were accumulated mainly in 9–15 DPA fibers, indicating they may participate in fiber development at rapid cell elongation stage. Besides, high expression levels of *GhbHLH020/102/256/351/354* genes were only found in 18–21 DPA fibers, implying that they may function in regulating secondary cell wall biosynthesis and deposition during cotton fiber development. In total, the above results suggested that GhbHLH/HLH transcription factors may play important roles in regulating cotton fiber development possibly through modulating BR signaling in fiber cells.

### GhbHLH282 plays a role in regulating plant growth by participating in response to BR signaling

**Fig. 7:**
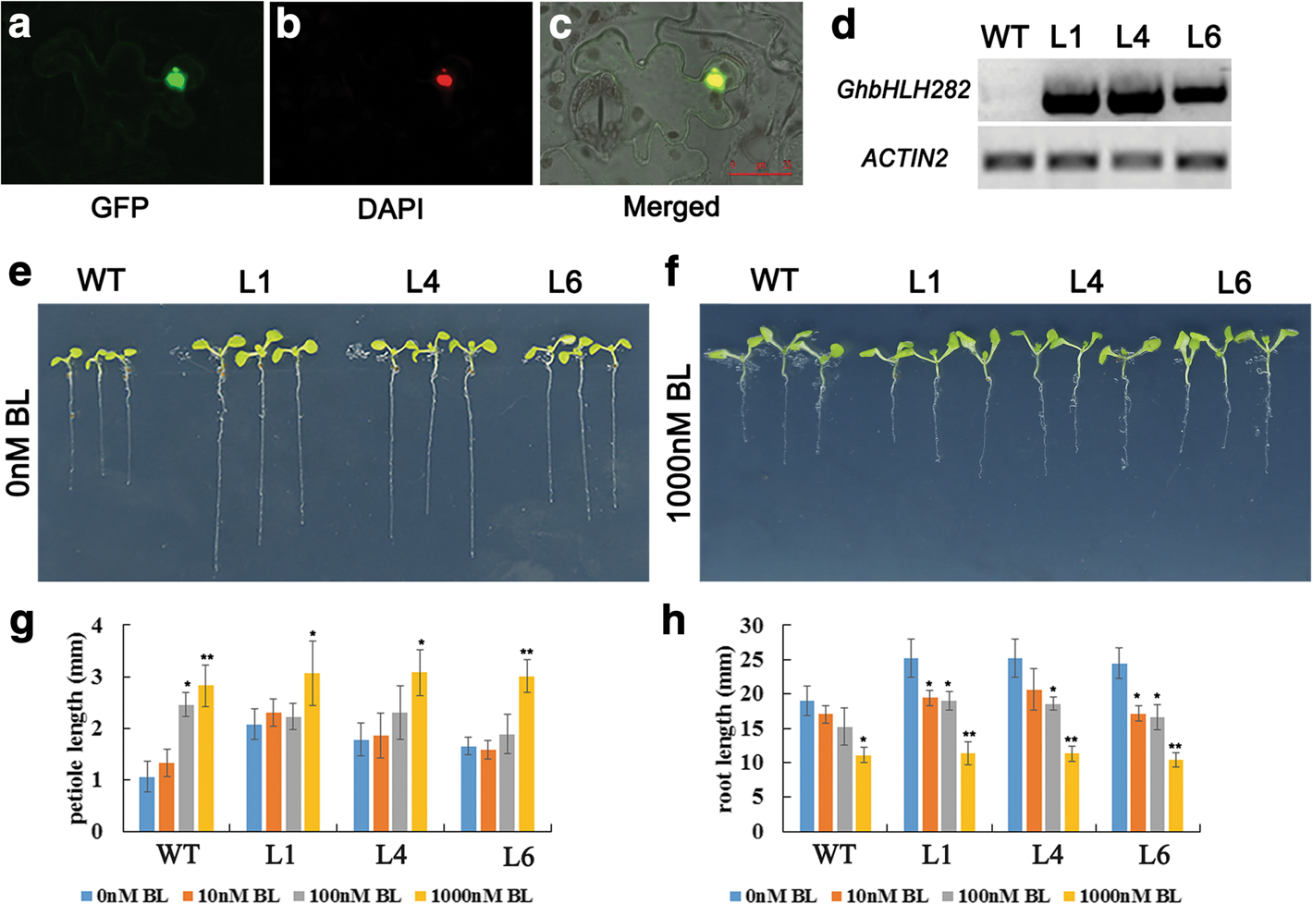
Phenotypic analysis of GhbHLH282-overexpressing transgenic Arabidopsis plants in responses to 2, 4-epibrassinolide (BL). **a-c** Subcellular localization of GhbHLH282 protein. **a** Fluorescence of eGFP:GhbHLH282 fusion proteins in the tobacco (*Nicotiana Benthamiana*) foliar epidermis cells. **b** Nuclear DAPI staining of the same cell in a. **c** The images of a and b were merged over the bright-field. **d** Semiquantitative RT-PCR analysis of *GhbHLH282* expression in the transgenic plants and wild type controls. Transcript levels of *GhbHLH282* were determined by Semiquantitative RT-PCR analysis, using *AtACTIN2* as a quantification control. **e** The phenotypes of *GhbHLH282*-overexpressing seedlings and wild type grown on half-strength MS medium without BL for 7 days. **f** The phenotypes of *GhbHLH282*-overexpressing seedlings and wild type grown on half-strength MS medium with 1000 nM BL for 7 days. **g** Statistical analysis of petiole length of the *GhbHLH282*-overexpressing transgenic lines and wild type. The petiole growth of *GhbHLH282*-overexpressing plants showed the decreased sensitivity to BL, compared with mock control (0 nM BL), when treated with 10, 100 and 1000 nM BL. **h** Statistical analysis of root length of the *GhbHLH282*-overexpressing transgenic lines and wild type. The root growth of *GhbHLH282*-overexpressing plants showed the increased sensitivity to BL, compared with mock control (0 nM BL), when treated with 10, 100 and 1000 nM BL. Mean values and standard deviations are shown from three independent experiments. One or two asterisks represent there was significant (*P* < 0.05) or very significant (P < 0.01) difference in petiole length or root length between the BL-treated sample and the untreated sample (control), respectively. WT, wild type; L1, L4 and L6, three *GhbHLH282* transgenic lines

To further investigate the roles of GhbHLH/HLH transcription factors in cotton fiber development, we chose *GhbHLH282* as a candidate gene that is preferentially expressed in 9–15 DPA fibers of cotton and may be related to BR signaling. Firstly, we assayed the subcellular localization of GhbHLH282 protein by employing the eGFP marker (GhbHLH282:eGFP fusion, see Methods). As shown in Fig. 7a, b and c, both the GFP fluorescence and DAPI staining were strongly accumulated in nuclei of tobacco foliar epidermis cells, suggesting that GhbHLH282 proteins were localized in the cell nucleus. Furthermore, we generated transgenic Arabidopsis plants overexpressing *GhbHLH282* gene, and selected three lines (GhbHLH282OX-1, 4 and 6) with different expression levels for further study. RT-PCR analysis revealed that *GhbHLH282* was expressed in the three transgenic lines and its transcripts were not detected in wild type (Fig.7d). It has been reported that BRs regulate root growth in a dose-dependent manner [17]. BRs also promoted the growth of petioles and cotyledons [24]. After 7 days incubation under light, we observed the phenotypes of the *GhbHLH282* transgenic lines and wild type. The transgenic seedlings showed longer petioles and roots compared with wild type controls (Fig.7e). Statistical analysis indicated that petiole length of GhbHLH282OX transgenic seedlings was significantly increased by approximately one-fold, and root length of the transgenic lines was significantly increased by nearly 40% relative to those of wild type (Fig.7g and h). When treated with 2,4-epibrassinolide (BL), the length of petioles was increased, whereas the length of roots was reduced in both wild type and the transgenic lines. When the concentration of BL reached 1000 nM, there was no significant phenotypic difference between the transgenic lines and wild type (Fig.7f). These results indicated that overexpression of GhbHLH282 in Arabidopsis rendered plants insensitive to BR-induced petioles growth promotion (Fig.7g). On the contrary, overexpression of GhbHLH282 also rendered plants hyposensitive to BR-induced root growth inhibition (Fig.7h). Besides, we also observed the hypocotyls of the transgenic plants and wild type under the dark. We found that dark-grown GhbHLH282OX seedlings didn’t show significant difference with wild type. Collectively, the above data suggested that GhbHLH282 may play different roles in BR-promoted petioles growth and BR-inhibited roots growth.

## Discussion

It is quite difficult to identify all the bHLH/HLH protein genes completely in a certain genome, especially in allotetraploid upland cotton (*G. hirsutum*) genome. Besides, the diversity and complexity of *bHLH/HLH* gene families caused the imperfection of *GhbHLH/HLH* genes identification. In previous study, a set of cotton *bHLH/HLH* reference genes containing 289 paralogs were identified from annotated genomes of *G. raimondii* and *G. arboreum* [37]. In our present study, we identified 437 *bHLH/HLH* transcription factor genes from just-completed allotetraploid *G. hirsutum* genome annotation, including 205 genes in A-subgenome and 232 genes in D-subgenome. The total number of *GhbHLH/HLH* genes is larger than that of Arabidopsis (167) and rice (177) reported [34]. The number of *bHLH/HLH* genes in upland cotton is nearly 2.6 times of that in Arabidopsis, which is consistent with the fact that the protein coding genes (66,434 genes) in cotton genome is about 2.6 times of that in Arabidopsis (25,498 genes) [32, 38]. It is found that many *bHLH/HLH* genes in Arabidopsis have two or more counterparts in upland cotton, indicating that the amplification of *bHLH/HLH* genes in upland cotton may be caused by gene duplication events. Previous studies reported that the expansion of gene families is probably due to gene duplication events, consisting of tandem duplication, segmental duplication, whole genome duplication and transposition events [39, 40]. Our results suggested that the segmental duplication may be a major duplication event and contribute most to the amplification of *bHLH/HLH* genes in upland cotton.

Brassinosteroid (BR) is not only involved in seed germination, vascular development and senescence of tissues, but also related to cell expansion and division [41–44]. For example, BIL4 regulates cell elongation via modulating BRI1 localization for BR signal transduction [45]. Exogenous BR application inhibits root elongation and promotes the lateral root initiation [46, 47]. Besides, in vitro studies suggested that pollen tube elongation could depend partly on BR [48]. Unlimitedly, BR is also required for normal cotton fiber cell development [26, 27]. A recent study indicated that BR signaling promotes fiber maturation of cotton through cellulose deposition in secondary cell walls [49]. *PAG1*, a cotton brassinosteroid catabolism gene, modulates fiber elongation via controlling the level of endogenous bioactive BR [50]. Also, our previous study revealed that Gh14–3-3 proteins regulate fiber initiation and elongation by modulating BR signaling [28]. These data indicate BR signaling plays an essential role in fiber development of cotton.

It has been reported that bHLH/HLH proteins as important regulators participate in BR signaling pathway. For instance, AtBIM2 participates in BR signaling by mediating the BR-regulated genes expression [51]. AtAIF4 is a negative regulator of BR signaling. Overexpression of *AtAIF4* results in dwarf transgenic plants, resembling BR mutants [16]. AtPREs and AtCIB3/5 are positive regulators in promoting cell elongation by modulating BR signaling [23, 24]. Besides, AtbHLH001 (GL3) and AtbHLH002 (EGL3) positively regulate the initiation of trichomes in Arabidopsis [11, 52]. In this study, expression of *GhbHLH200* was induced by BL treatment, and its transcripts were accumulated in 6–18 DPA fibers, indicating it is probably involved in cotton fiber elongation. Expression of *GhbHLH109* and *GhbHLH319* was inhibited by BL and induced by Brz, and its transcripts were predominantly accumulated in very early developing ovules and fibers, suggesting that these genes may have important roles in fiber initiation. Furthermore, the transcripts of *GhbHLH282* were accumulated mainly in 9–15 DPA fibers, and its expression was inhibited by BL but induced by Brz. Overexpressing GhbHLH282 not only promoted plant growth, but also changed plant sensitivity to BL signaling, suggesting that GhbHLH282 may play an important role in regulating cotton fiber development via BR signaling pathway. Collectively, we identified 59 BR responsive *GhbHLH/HLH* genes, which are differentially expressed during fiber initiation, elongation and secondary cell wall biosynthesis stages, implying these *GhbHLH/HLH* genes may participate in response to BR signaling for regulating cotton fiber development.

## Conclusions

In summary, we performed a systematic analysis of the *bHLH/HLH* gene family in upland cotton (*G. hirsutum*) genome, including gene classification, phylogenetic relationship, chromosomal distribution, gene expansion, gene structure, and motif composition, as well as gene expression pattern in fiber development and in response to BR signaling. Furthermore, our results revealed the role of GhbHLH282 in plant growth possibly via BR signaling pathway. Thus, the data reported here may facilitate a more comprehensive understanding of the specific roles of the *bHLH/HLH* genes in fiber development of cotton.

## Methods

### Plant materials

Upland cotton (*Gossypium hirsutum*) cultivar Coker312 was used in this study. Cotton plants grew in the trial field located at campus of Cental China Normal University, Wuhan, China. Flowers were tagged on the day of anthesis. Cotton bolls were harvested from the plants at 0, 3, 6, 9, 12, 15, 18 and 21 days post anthesis (DPA), respectively. Ovules and fibers at different stages were removed from the collected bolls carefully. All collected materials were frozen immediately in liquid nitrogen and stored at − 80 °C until RNA extraction.

*Arabidopsis thaliana* Columbia (Col-0) ecotype was used as the wild type for generating the transgenic plants. Seven-day-old seedlings grown on a half-strength MS agar medium were used as the experimental materials.

Both cotton and Arabidopsis seeds used in this study were provided by our lab.

### Identification of GhbHLH/HLH transcription factors

To identify *bHLH/HLH* genes in upland cotton, all published *bHLH/HLH* gene sequences of Arabidopsis and rice were employed as queries to perform homologous blast searches against the upland cotton (*G. hirsutum*) genome databases (https://www.cottongen.org/tools/blast/blast) [32]. Furthermore, the conserved domains of predicted bHLH/HLH proteins were evaluated using the hmmscan program and the pfam tools (http://www.ebi.ac.uk/Tools/hmmer/search/hmmscan) according to reported methods [33].

### Phylogenetic analysis

Multiple sequence alignments were conducted on the conserved bHLH/HLH domains of the identified bHLH/HLH protien sequences in upland cotton, Arabidopsis and rice genomes by Muscle aligin method using MEGA 6.0 software. Based on the results of multiple sequence alignment, MEGA 6.0 software was employed to construct an unrooted phylogenetic tree based on alignments using the NeighborJoining (NJ) method with the following parameters: No. of differences model, pairwise deletion and 1000 bootstraps [53]. Additionally, a separate phylogenetic tree was constructed with all the conserved domains of GhbHLH/HLH protein sequences for further analysis. Then the unrooted phylogenetic tree was subjected to ITOL (http://itol.embl.de/upload.cgi) to form the interactive tree.

### Analysis of chromosomal distribution and duplication of genes

To get the information about the physical locations of all *GhbHLH/HLH* genes on chromosomes, blastn searches against upland cotton (*G. hirsutum*) genome databases (https://www.cottongen.org/tools/blast/blast) were employed. Then all the *GhbHLH/HLH* genes were mapped on the chromosomes using CorelDRAW ×7 software except for the scaffolds genes. The *GhbHLH/HLH* gene duplication events were detected according to the criteria described in previous studies [54, 55]. Paralogous *bHLH/HLH* gene pairs were obtained based on alignment results.

### Assay of gene structure and conserved motifs

The *GhbHLH/HLH* gene sequences and CDS sequences were identified from upland cotton (*G. hirsutum*) genome databases described above and were loaded into gene structure display server program (http://gsds.cbi.pku.edu.cn/) to infer the exon/intron organization of *bHLH/HLH* genes. To identify the conserved protein motifs, the bHLH/HLH protein sequences were submitted to online Multiple Expectation maximization for Motif Elicitation (http://meme-suite.org/tools/meme) program. The optimized MEME parameters are as follows: any number of repetitions, the optimum width from 6 to 100. The identified protein motifs were further annotated with ScanProsite.

### Analysis of gene expression based on transcriptome data

To uncover the *GhbHLH/HLH* gene expression patterns in cotton different tissues, the RPKM (reads per kb per million reads) values which denoted the expression levels of *bHLH/HLH* genes were obtained from a comprehensive profile of the TM-1 transcriptome data (Accession codes, SRA: PRJNA248163, http://www.ncbi.nlm.nih.gov/sra/?term=PRJNA248163).

### In vitro culture of cotton ovules

Bolls at 9 DPA (day post anthesis) from cotton plants were surface sterilized with 70% ethanol for 1 min, followed by washing with sterile water. Ovules were picked out from these sterilized bolls, and cultured in BT liquid medium containing 5 μM NAA and 0.5 μM GA3 [56], supplemented with 0 nM (control), 10 nM, 100 nM and 1000 nM 2,4-epibrassinolide (BL) or brassinazole2001 (Brz) at 30 °C in dark for 3 h. Then Ovules and fibers were removed from the collected bolls carefully. All collected materials were frozen immediately in liquid nitrogen and stored at − 80 °C until RNA extraction. Experiments were repeated at least three times and ran in three replicates each time.

### RNA isolation and RT-PCR analysis

Total RNA was extracted from collected materials using Tiangen RNAprep Pure Plant Kit according to the manufacturer’s instructions. The total of 2 μg RNA was employed as the template for the synthesis of cDNA first-strands using M-MLV reverse transcriptase (Promega, Madison, WI) according to the manufacturer’s instructions. Gene expression levels were analyzed by real-time PCR using the fluorescent intercalating dye SYBR-Green in a detection system. The expression values of *bHLH/HLH* genes tested were normalized with an internal reference polyubiquitin gene (*GhUBI1*, access number in GenBank: EU604080). The relative expression levels (R) were calculated using the following equation: *R* = 2^-(Ct1-Ct2)^, where Ct1 refers to the Ct value of *bHLH/HLH* genes while Ct2 is the Ct value of the reference gene. RT-PCR data are mean values and standard deviations (bar) of three independent experiments with three biological replicates. Based on the values of relative expression levels, heatmaps for gene expression patterns were generated by the online program Omicshare (http://www.omicshare.com/tools/Home/Soft/heatmap).

Gene expression levels in wild type and transgenic Arabidopsis were analyzed by semiquantitative RT-PCR analysis using the Hieff™ PCR Master Mix in 96 Well Thermal Cycler (Applied Biosystems). The expression levels of *GhbHLH282* gene were tested after cDNAs were normalized with an internal reference gene Arabidopsis *ACTIN2* (AF428330).

### Generation of transgenic Arabidopsis plants

The coding sequence of *GhbHLH282* was PCR-amplified using proofreading Pfu DNA polymerase and subsequently subcloned into the expression vector *pCAMBIA-2300* under the control of the Caulifower mosaic virus *(CaMV) 35S* promoter. The recombinant vector construct was transferred into *Agrobacterium tumefaciens* GV3101 and then introduced into Arabidopsis using the floral dipping method. The transformed Arabidopsis seeds were selected on MS medium containing 50 mg/L kanamycin. Homozygous lines of T3 generations were used for phenotypic analysis.

### Subcellular localization of GhbHLH282 protein

The coding sequence of *GHbHLH282* was cloned into the downstream region of *eGFP* in the expression vector *pCAMBIA-2300-35S-eGFP* under the control of the Caulifower mosaic virus *(CaMV) 35S* promoter. The recombinant vector *pCAMBIA-2300-35S-eGFP-GhbHLH282* was transferred into *Agrobacterium tumefaciens* GV3101 and then introduced into fully expanded leaves of tobacco (*Nicotiana Benthamiana*) plants using a needleless syringe. After infiltration, plants were immediately covered with plastic bags and placed at 23 °C for 48 h, and then incubated at 28 °C under a photoperiod of 16 h light/8 h dark. Tobacco foliar epidermis were stained with 4′6-diamidino-2-phenylindole (DAPI, a nucleus-specific dye) for 1 min at room temperature before observation of GFP fluorescence and DAPI staining under the confocal fluorescence microscope (Leica, Germany). The digital images were taken and processed by SP5 software (Leica, Germany).

### Measurement and statistical analysis

Arabidopsis seedlings grown on a half-strength MS medium in the presence or absence of the indicated concentrations of 2,4-epibrassinolide (BL) under a photoperiod of 16 h light/8 h dark at 22 °C were photographed, and petiole and root length of these seedlings was measured using the Image J software (https://imagej.en.softonic.com/). All experiments were done at a minimum in triplicate, and the data were statistically analyzed by the Student’s t-test. More than 50 seedlings were used for each biological replicate.

All primers used are listed in Additional file 1: Table S3.

## Additional files

Additional file 1: **Table S1.** The *bHLH/HLH* gene family of cotton (*Gossypium hirsutum*). **Figure S1.** Phylogenetic relationship of cotton (*Gossypium hirsutum*) bHLH/HLH transcription factors with those of *Arabidopsis* and rice. **Table S2.** Phylogenetic classification and known biological functions of bHLH/HLH proteins from Arabidopsis and cotton (*Gossypium hirsutum*). **Figure S2.** Conserved motifs of GhbHLH/HLH proteins. **Figure S3.** Heatmap representation for expression profiles of cotton (*G. hirsutum*) *bHLH/HLH* genes in cotton tissues. **Table S3.** Primers used in quantitative RT-PCR analysis. (PDF 704 kb)
