# Supplementary material for: Characterization of bHLH/HLH genes that are involved in brassinosteroid (BR) signaling in fiber development of cotton (Gossypium hirsutum)
Source: BMC Plant Biol. 2018 Nov 27;18:304. doi: 10.1186/s12870-018-1523-y (PMC6258498; doi:10.1186/s12870-018-1523-y)
Supplement: Supplementary file 1 — Table S1. The bHLH/HLH gene family of cotton (Gossypium hirsutum). Figure S1. Phylogenetic relationship of cotton (Gossypium hirsutum) bHLH/HLH transcription factors with those of Arabidopsis and rice. Table S2. Phylogenetic classification and known biological functions of bHLH/HLH proteins from Arabidopsis and cotton (Gossypium hirsutum). Figure S2. Conserved motifs of GhbHLH/HLH proteins. Figure S3. Heatmap representation for expression profiles of cotton (G. hirsutum) bHLH/HLH genes in cotton tissues. Table S3. Primers used in quantitative RT-PCR analysis. (PDF 704 kb) [file 12870_2018_1523_MOESM1_ESM.pdf]

Additional file 1

Table S1. ***bHLH/HLH*** **gene family of cotton (*Gossypium hirsutum*)**

Gene Name	Gene symbol	length(aa)	MW(Da)	pI	Chr location
GhbHLH001	Gh_A01G0544	474	52093.67	7.199	A01:9100896..9102924
GhbHLH002	Gh_A01G0566	238	26175.67	7.189	A01:9991487..9994331
GhbHLH003	Gh_A01G0772	254	29134.53	5.908	A01:16400214..16401057
GhbHLH004	Gh_A01G1034	323	37144.2	5.798	A01:32881413..32882614
GhbHLH005	Gh_A01G1502	346	38952.99	7.319	A01:90754508..90755845
GhbHLH006	Gh_A01G1661	346	37901.3	8.914	A01:95117537..95119757
GhbHLH007	Gh_A01G1663	361	39733.38	5.968	A01:95183534..95187618
GhbHLH008	Gh_A01G2041	231	25590.33	7.673	scaffold32_A01:115876..117678
GhbHLH009	Gh_A02G0164	441	48276.94	6.119	A02:1745614..1747359
GhbHLH010	Gh_A02G0343	234	27228.53	9.205	A02:4056763..4057616
GhbHLH011	Gh_A02G0792	326	36682.49	6.408	A02:16373680..16374849
GhbHLH012	Gh_A02G0958	842	92266.33	5.078	A02:40584297..40588366
GhbHLH013	Gh_A02G1131	423	46234.68	5.145	A02:61707065..61708336
GhbHLH014	Gh_A02G1241	323	35943.52	6.851	A02:73602063..73603221
GhbHLH015	Gh_A02G1404	336	38108.09	5.231	A02:79623484..79624741
GhbHLH016	Gh_A02G1414	359	39890.11	7.436	A02:79815325..79817184
GhbHLH017	Gh_A02G1415	323	36554.32	6.062	A02:79849328..79850543
GhbHLH018	Gh_A02G1502	227	25758.13	9.162	A02:81211553..81212236
GhbHLH019	Gh_A02G1636	245	27646.12	8.577	A02:82809153..82810922
GhbHLH020	Gh_A03G0010	549	61007.38	5.973	A03:165999..167648
GhbHLH021	Gh_A03G0490	554	60699.66	5.958	A03:11197253..11200443
GhbHLH022	Gh_A03G0577	91	10359.67	8.938	A03:15058399..15058983
GhbHLH023	Gh_A03G0607	419	46264.79	6.766	A03:16811556..16813669
GhbHLH024	Gh_A03G0837	184	20728.99	9.537	A03:45046677..45048140
GhbHLH025	Gh_A03G0864	93	10558.97	9.843	A03:50191438..50192040
GhbHLH026	Gh_A03G0937	580	62732.86	7.664	A03:60100877..60103638
GhbHLH027	Gh_A03G1273	248	27542.92	7.397	A03:89113314..89115198
GhbHLH028	Gh_A03G1436	319	36055.53	7.06	A03:94233183..94235026
GhbHLH029	Gh_A03G1607	271	29841.19	4.632	A03:97243906..97245079
GhbHLH030	Gh_A03G1661	129	14962.44	7.039	A03:97784185..97785285
GhbHLH031	Gh_A03G1793	345	38214.62	6.368	A03:99074853..99077771
GhbHLH032	Gh_A03G2155	270	30694.01	6.416	scaffold734_A03:42967..44448
GhbHLH033	Gh_A03G2173	284	31856.66	7.388	scaffold738_A03:6061..7550
GhbHLH034	Gh_A04G0201	388	43050.93	7.48	A04:4062775..4065919
GhbHLH035	Gh_A04G0695	111	12624.47	9.24	A04:46784834..46785308
GhbHLH036	Gh_A04G0803	171	19075.58	8.415	A04:53453230..53453745
GhbHLH037	Gh_A04G0856	275	30681.03	6.692	A04:55816183..55817966
GhbHLH038	Gh_A04G0887	235	27305.31	7.006	A04:56956093..56956962
GhbHLH039	Gh_A04G1091	323	36807.66	7.69	A04:60971671..60973377
GhbHLH040	Gh_A05G0019	218	24459.25	7.963	A05:276521..277548

GhbHLH041	Gh_A05G0224	561	63055.73	5.634	A05:2426716..2428994
GhbHLH042	Gh_A05G0268	502	53967.47	4.924	A05:3131917..3134242
GhbHLH043	Gh_A05G0416	250	26068.44	8.911	A05:4697569..4700249
GhbHLH044	Gh_A05G0559	624	68143.81	6.745	A05:5978961..5981335
GhbHLH045	Gh_A05G0570	263	29647.87	6.096	A05:6128505..6129489
GhbHLH046	Gh_A05G0640	859	94387.17	5.838	A05:6755868..6760478+
GhbHLH047	Gh_A05G0723	322	36263.13	4.75	A05:7525898..7527188
GhbHLH048	Gh_A05G1310	277	30635.85	7.924	A05:13448825..13451409
GhbHLH049	Gh_A05G1422	466	51013.85	5.463	A05:14764181..14766496
GhbHLH050	Gh_A05G1429	253	27769.42	5.941	A05:14810318..14811765
GhbHLH051	Gh_A05G1755	269	29672.88	7.598	A05:18522791..18523600
GhbHLH052	Gh_A05G1764	333	35656.63	5.666	A05:18608873..18611972
GhbHLH053	Gh_A05G1820	376	41407.97	4.697	A05:19097003..19098446
GhbHLH054	Gh_A05G1878	230	25551	8.9	A05:19676115..19676892
GhbHLH055	Gh_A05G1905	337	37331.12	6.05	A05:20004376..20005794
GhbHLH056	Gh_A05G2270	1001	113945.05	4.976	A05:26636061..26640061
GhbHLH057	Gh_A05G2273	356	38854.34	4.872	A05:26703079..26704149
GhbHLH058	Gh_A05G2637	93	10570.09	4.388	A05:40737191..40737631
GhbHLH059	Gh_A05G2652	328	36389.2	6.07	A05:41824187..41826793
GhbHLH060	Gh_A05G2888	248	28059.49	6.617	A05:68355802..68356804
GhbHLH061	Gh_A05G3639	447	50822.01	8.471	scaffold1095_A05:51690..54441
GhbHLH062	Gh_A05G3762	340	38760.92	4.583	scaffold1216_A05:98122..99419
GhbHLH063	Gh_A05G3795	433	47850.6	6.904	scaffold1225_A05:36332..39452
GhbHLH064	Gh_A05G3957	333	36065.95	7.286	scaffold1244_A05:15623..17735
GhbHLH065	Gh_A06G0057	342	37887.38	9.118	A06:481200..484598
GhbHLH066	Gh_A06G0161	204	22563.08	9.285	A06:1664199..1664813
GhbHLH067	Gh_A06G0223	413	44841.09	6.34	A06:2537948..2539953
GhbHLH068	Gh_A06G0241	331	37714.65	4.571	A06:2939327..2941225
GhbHLH069	Gh_A06G0553	113	12453.99	5.008	A06:14187167..14189713
GhbHLH070	Gh_A06G0646	685	77577.44	9.466	A06:16998718..17001346
GhbHLH071	Gh_A06G0872	427	45549.17	6.852	A06:33287675..33290588
GhbHLH072	Gh_A06G1395	430	47535.33	5.687	A06:96198720..96200266
GhbHLH073	Gh_A06G1396	428	47520.26	5.683	A06:96220925..96222465
GhbHLH074	Gh_A06G1858	273	30428.97	5.3	scaffold1254_A06:83045..84473
GhbHLH075	Gh_A06G1891	406	44599.25	4.763	scaffold1263_A06:56713..58302
GhbHLH076	Gh_A06G2080	178	20301.86	8.928	scaffold1440_A06:13274..14056
GhbHLH077	Gh_A07G0112	439	48950.91	9.6	A07:1363930..1367363
GhbHLH078	Gh_A07G0148	487	52047.35	4.762	A07:1859863..1862046
GhbHLH079	Gh_A07G0414	294	32454.99	9.399	A07:5307473..5310487
GhbHLH080	Gh_A07G0598	275	30857.58	6.086	A07:8284166..8285748
GhbHLH081	Gh_A07G0629	432	47559.61	8.514	A07:8819787..8822516
GhbHLH082	Gh_A07G0692	294	32555.31	7.419	A07:10204458..10206548
GhbHLH083	Gh_A07G1202	473	51163.31	8.927	A07:26807317..26809224
GhbHLH084	Gh_A07G1232	502	56093.49	5.188	A07:28102807..28104315

GhbHLH085	Gh_A07G1552	120	13142.4	4.569	A07:56194285..56196990
GhbHLH086	Gh_A07G1668	145	16705.53	10.604	A07:67649445..67662845
GhbHLH087	Gh_A07G1800	303	34001	6.608	A07:72847192..72848543
GhbHLH088	Gh_A07G1843	526	57658.48	5.856	A07:73428377..73432383
GhbHLH089	Gh_A07G1925	732	80645.09	6.073	A07:74990646..74996538-
GhbHLH090	Gh_A07G1964	94	10463.9	8.203	A07:75596676..75598867
GhbHLH091	Gh_A07G2140	486	53433.63	5.792	A07:78095014..78098747
GhbHLH092	Gh_A07G2157	299	31782.21	8.166	scaffold1615_A07:21601..23685
GhbHLH093	Gh_A07G2268	508	55412.2	7.18	scaffold1903_A07:71709..75259
GhbHLH094	Gh_A07G2286	247	27408.88	7.459	scaffold1913_A07:12876..13866
GhbHLH095	Gh_A08G0230	339	38079.67	7.153	A08:2450729..2452961
GhbHLH096	Gh_A08G0554	733	80620.35	6.625	A08:8664775..8670632+
GhbHLH097	Gh_A08G0962	462	51739.77	7.866	A08:64469861..64471249
GhbHLH098	Gh_A08G0969	818	89794.73	5.797	A08:66477394..66481360
GhbHLH099	Gh_A08G1091	476	53169.98	6.683	A08:75530415..75531845
GhbHLH100	Gh_A08G1412	674	73570.89	5.508	A08:89468169..89470193
GhbHLH101	Gh_A08G1636	435	49159.64	5.711	A08:95725449..95731601
GhbHLH102	Gh_A08G1678	305	32119.25	5.714	A08:96441433..96444057
GhbHLH103	Gh_A08G1880	331	37202.87	7.168	A08:99495521..99496694
GhbHLH104	Gh_A08G2365	679	75979.95	4.977	scaffold1936_A08:18100..24216
GhbHLH105	Gh_A08G2484	304	31985.21	6.463	scaffold2257_A08:19112..22894
GhbHLH106	Gh_A08G2486	255	28439.81	6.241	scaffold2258_A08:22227..23256
GhbHLH107	Gh_A09G0075	374	41992.48	5.629	A09:1740565..1744247
GhbHLH108	Gh_A09G0190	93	10540.6	9.548	A09:5441227..5441589
GhbHLH109	Gh_A09G0192	92	10382.84	8.205	A09:5625680..5626482
GhbHLH110	Gh_A09G0477	268	30438.21	8.251	A09:38007992..38009617
GhbHLH111	Gh_A09G0522	192	21325.89	7.734	A09:42088394..42090283
GhbHLH112	Gh_A09G0675	347	38168.39	7.716	A09:52024537..52027545
GhbHLH113	Gh_A09G0939	921	100062.77	5.861	A09:58447731..58453968-
GhbHLH114	Gh_A09G0994	205	23544.3	8.905	A09:59857537..59858236
GhbHLH115	Gh_A09G1174	374	41014.06	6.366	A09:63250034..63255154
GhbHLH116	Gh_A09G1177	522	57668.9	7.835	A09:63275373..63282824
GhbHLH117	Gh_A09G1589	282	31872.51	6.765	A09:69307505..69308848
GhbHLH118	Gh_A09G1600	229	25719.18	7.394	A09:69566712..69567670
GhbHLH119	Gh_A09G1777	295	33296.37	9.075	A09:71093227..71095013
GhbHLH120	Gh_A09G1863	142	15922.69	9.839	A09:71872984..71874868
GhbHLH121	Gh_A09G1952	227	26300.15	9.698	A09:72626500..72628986
GhbHLH122	Gh_A09G2341	659	71540.72	5.55	scaffold2291_A09:148726..150705
GhbHLH123	Gh_A09G2474	309	34862.49	4.688	scaffold2314_A09:10661..12256
GhbHLH124	Gh_A10G0164	222	25580.51	6.516	A10:1446697..1448045
GhbHLH125	Gh_A10G0166	351	38624.23	5.801	A10:1464534..1466486
GhbHLH126	Gh_A10G0292	257	28796.81	7.82	A10:2655630..2657664
GhbHLH127	Gh_A10G0560	413	45365.35	5.294	A10:6916375..6917616
GhbHLH128	Gh_A10G0730	328	36020.47	6.422	A10:12803714..12806410

GhbHLH129	Gh_A10G0750	223	25232.81	6.841	A10:13837850..13838521
GhbHLH130	Gh_A10G1013	212	24237.4	7.304	A10:30110451..30112591
GhbHLH131	Gh_A10G1098	414	47148.15	5.661	A10:52020562..52021983
GhbHLH132	Gh_A10G1164	517	56294.99	7.057	A10:59724810..59726890
GhbHLH133	Gh_A10G1210	340	36629.93	9.27	A10:63248137..63253499
GhbHLH134	Gh_A10G1280	150	16844.32	9.028	A10:66983133..66984953
GhbHLH135	Gh_A10G1739	713	80627.62	8.646	A10:91472268..91491746
GhbHLH136	Gh_A10G2158	238	26271.03	8.525	A10:100443417..100444989
GhbHLH137	Gh_A10G2207	186	20877.52	8.469	scaffold2446_A10:53829..54551
GhbHLH138	Gh_A11G0202	629	70546.86	5.738	A11:1936618..1939038
GhbHLH139	Gh_A11G0278	216	23815.92	7.43	A11:2565830..2567110
GhbHLH140	Gh_A11G0318	513	56654.31	9.121	A11:2918643..2928372
GhbHLH141	Gh_A11G0353	332	37143.85	8.255	A11:3241409..3243131
GhbHLH142	Gh_A11G0357	564	62236.59	6.74	A11:3290744..3293533
GhbHLH143	Gh_A11G0459	302	34149.58	6.141	A11:4401426..4403145
GhbHLH144	Gh_A11G0468	210	24155.37	10.385	A11:4513470..4514654
GhbHLH145	Gh_A11G0588	520	56503.63	5.361	A11:5619172..5622722
GhbHLH146	Gh_A11G0710	472	51283.56	8.091	A11:6911332..6914629
GhbHLH147	Gh_A11G0796	631	71270.91	5.349	A11:7913452..7916208
GhbHLH148	Gh_A11G0884	616	68122.43	6.551	A11:9013589..9015439
GhbHLH149	Gh_A11G0909	495	55189.49	5.376	A11:9593471..9594958
GhbHLH150	Gh_A11G0922	414	47294.66	6.362	A11:9799368..9856355
GhbHLH151	Gh_A11G1115	338	37880.22	4.856	A11:13055890..13058079
GhbHLH152	Gh_A11G1120	627	70694.18	5.913	A11:13156892..13161142
GhbHLH153	Gh_A11G1163	303	32009.25	6.465	A11:14172674..14176532
GhbHLH154	Gh_A11G1180	504	55686.9	5.525	A11:14407742..14409256
GhbHLH155	Gh_A11G1248	548	59921.69	6.885	A11:15529412..15532654
GhbHLH156	Gh_A11G1301	547	59543.61	6.858	A11:16746579..16748918
GhbHLH157	Gh_A11G1614	279	30979.56	5.21	A11:23972119..23973936
GhbHLH158	Gh_A11G1781	394	43324.35	5.431	A11:31338753..31341621
GhbHLH159	Gh_A11G1886	349	39513.64	4.914	A11:48941582..48942891
GhbHLH160	Gh_A11G1894	328	36985.88	5.999	A11:49857411..49858778
GhbHLH161	Gh_A11G1897	362	40084.14	7.066	A11:50365734..50367088
GhbHLH162	Gh_A11G2243	325	33971.52	5.962	A11:77247831..77251718
GhbHLH163	Gh_A11G2386	314	34895.08	6.254	A11:81625266..81626383
GhbHLH164	Gh_A11G2494	678	73057.49	6.022	A11:83670084..83673290
GhbHLH165	Gh_A11G2537	237	26362.75	5.936	A11:84373515..84376323
GhbHLH166	Gh_A11G2746	233	26189.88	7.815	A11:90085542..90087830
GhbHLH167	Gh_A11G2752	228	25611.14	6.237	A11:90198561..90200028
GhbHLH168	Gh_A11G2808	361	39771.38	6.121	A11:91436486..91442266
GhbHLH169	Gh_A11G2956	235	27213.37	5.306	scaffold2723_A11:67019..68759
GhbHLH170	Gh_A11G2978	285	30618.08	5.99	scaffold2726_A11:30520..33385
GhbHLH171	Gh_A11G3000	92	10419.69	8.224	scaffold2728_A11:18718..19750
GhbHLH172	Gh_A11G3067	564	62686.52	7.546	scaffold2743_A11:33938..36126

GhbHLH173	Gh_A12G0259	448	49668.26	6.212	A12:4182812..4185230
GhbHLH174	Gh_A12G0337	602	68014.3	5.276	A12:6176078..6178491
GhbHLH175	Gh_A12G0489	245	27821.62	7.138	A12:10837106..10838921
GhbHLH176	Gh_A12G0596	436	48192.28	7.01	A12:15032546..15035004
GhbHLH177	Gh_A12G0666	239	26501.93	6.613	A12:22835707..22837742
GhbHLH178	Gh_A12G0736	312	35331.05	6.408	A12:36986720..36987967
GhbHLH179	Gh_A12G1040	490	54818.32	5.289	A12:62193253..62194725
GhbHLH180	Gh_A12G1276	212	24892.54	8.504	A12:67769301..67770110
GhbHLH181	Gh_A12G1319	470	53491.41	8.568	A12:69691350..69693837
GhbHLH182	Gh_A12G1457	295	32785.27	8.341	A12:73807628..73810081
GhbHLH183	Gh_A12G1523	92	10344.7	9.584	A12:74963898..74964384
GhbHLH184	Gh_A12G1560	345	38262.37	6.111	A12:75726671..75728454
GhbHLH185	Gh_A12G1600	87	9729.03	5.121	A12:76818299..76818824
GhbHLH186	Gh_A12G1755	336	37401.01	6.741	A12:79661194..79663140
GhbHLH187	Gh_A12G1889	331	37499.23	8.353	A12:81507858..81510025
GhbHLH188	Gh_A12G1893	646	71086.44	5.641	A12:81570988..81572928
GhbHLH189	Gh_A12G2172	475	53045.76	6.626	A12:84554992..84556419
GhbHLH190	Gh_A12G2377	205	22752.87	5.575	A12:86248253..86253761
GhbHLH191	Gh_A12G2534	262	28897.32	6.247	scaffold3064_A12:135605..139065
GhbHLH192	Gh_A13G0192	142	16315.61	9.719	A13:2182124..2185707
GhbHLH193	Gh_A13G0291	428	47149.41	6.16	A13:3572698..3574345
GhbHLH194	Gh_A13G0307	361	40534.48	5.518	A13:3820588..3822955
GhbHLH195	Gh_A13G0361	266	29527.35	6.983	A13:4819541..4822307
GhbHLH196	Gh_A13G0389	249	26588.63	6.593	A13:5148255..5149298
GhbHLH197	Gh_A13G0641	89	9865.22	7.286	A13:17481549..17482231
GhbHLH198	Gh_A13G0762	327	36462.45	6.563	A13:32010000..32012589
GhbHLH199	Gh_A13G0768	1302	143238.61	7.248	A13:32111193..32116835
GhbHLH200	Gh_A13G0929	199	22580.2	11.358	A13:49726262..49726861
GhbHLH201	Gh_A13G1001	269	29280.66	6.426	A13:55612317..55616299
GhbHLH202	Gh_A13G1200	237	26212.71	6.427	A13:65044058..65046732
GhbHLH203	Gh_A13G1525	289	32601.06	8.528	A13:73818719..73819750
GhbHLH204	Gh_A13G1818	249	28349.2	5.206	A13:77767688..77773743
GhbHLH205	Gh_A13G2128	332	36288.12	6.867	scaffold3432_A13:65005..66950
GhbHLH206	Gh_D01G0152	231	25620.32	6.957	D01:1114918..1116682
GhbHLH207	Gh_D01G0554	475	52286.88	7.22	D01:7145827..7147867
GhbHLH208	Gh_D01G0575	238	26274.85	6.424	D01:7899356..7902320
GhbHLH209	Gh_D01G0798	260	29397.88	5.105	D01:12361920..12362780
GhbHLH210	Gh_D01G0879	73	8734.32	9.784	D01:14457120..14458334
GhbHLH211	Gh_D01G1057	254	28374.13	6.341	D01:19972710..19973979
GhbHLH212	Gh_D01G1087	323	37230.21	5.514	D01:21776648..21777837
GhbHLH213	Gh_D01G1748	346	39015.08	7.063	D01:54245946..54247158
GhbHLH214	Gh_D01G1910	346	37896.24	8.791	D01:57355612..57357824
GhbHLH215	Gh_D01G1913	361	39964.88	6.445	D01:57396146..57400300
GhbHLH216	Gh_D02G0116	155	17145.59	7.113	D02:939331..941543

GhbHLH217	Gh_D02G0205	441	48459.22	6.406	D02:2309366..2311112
GhbHLH218	Gh_D02G0406	234	27388.68	9.612	D02:5260298..5261153
GhbHLH219	Gh_D02G0668	87	10166.94	9.971	D02:9294403..9295898
GhbHLH220	Gh_D02G0841	340	38570.83	9.037	D02:14648518..14649628
GhbHLH221	Gh_D02G1179	184	20692.07	9.537	D02:35783317..35784763
GhbHLH222	Gh_D02G1245	93	10648.01	8.928	D02:39775325..39775925
GhbHLH223	Gh_D02G1321	526	56443.16	6.085	D02:43593015..43595420
GhbHLH224	Gh_D02G1714	248	27474.82	7.438	D02:58639784..58641658
GhbHLH225	Gh_D02G1895	320	36312.87	6.595	D02:62568751..62570417
GhbHLH226	Gh_D02G1995	271	30816.17	6.334	D02:63981726..63983210
GhbHLH227	Gh_D02G2021	272	29961.34	4.718	D02:64494542..64495692
GhbHLH228	Gh_D02G2076	133	15218.92	9.489	D02:64935755..64937177
GhbHLH229	Gh_D02G2226	345	38153.58	6.368	D02:66215043..66217869
GhbHLH230	Gh_D03G0086	315	35294.29	8.469	D03:609679..611835
GhbHLH231	Gh_D03G0212	227	25756.18	9.145	D03:2203285..2203968
GhbHLH232	Gh_D03G0304	324	36620.34	5.692	D03:3442396..3443617
GhbHLH233	Gh_D03G0305	359	40036.39	7.358	D03:3484532..3486295
GhbHLH234	Gh_D03G0317	337	38289.33	5.077	D03:3619141..3620402
GhbHLH235	Gh_D03G0375	321	35810.3	7.742	D03:4981728..4982882
GhbHLH236	Gh_D03G0554	423	46066.33	4.908	D03:10882630..10883901
GhbHLH237	Gh_D03G0792	842	92230.07	5.18	D03:27281464..27285533
GhbHLH238	Gh_D03G0859	91	10359.67	8.938	D03:29739821..29740474
GhbHLH239	Gh_D03G0895	419	46158.67	6.906	D03:30671870..30673972
GhbHLH240	Gh_D03G1047	512	56037.66	5.841	D03:35409534..35412590
GhbHLH241	Gh_D03G1849	549	60701.99	6.174	scaffold3940_D03:28887..30536
GhbHLH242	Gh_D04G0248	227	24888.17	8.884	D04:3756007..3756897
GhbHLH243	Gh_D04G0454	91	10281.7	8.221	D04:7304311..7304656
GhbHLH244	Gh_D04G0664	241	27197.41	5.1	D04:12878412..12879409
GhbHLH245	Gh_D04G0689	388	43281.63	5.773	D04:13557570..13559029
GhbHLH246	Gh_D04G1295	171	19075.58	8.415	D04:42505415..42505930
GhbHLH247	Gh_D04G1354	331	36606.63	6.534	D04:44158637..44161270
GhbHLH248	Gh_D04G1389	235	27196.18	7.422	D04:45074798..45075667
GhbHLH249	Gh_D04G1696	79	9193.38	5.263	D04:49051899..49052625
GhbHLH250	Gh_D04G1698	302	34003.21	7.017	D04:49076835..49078472
GhbHLH251	Gh_D04G1777	161	18302.57	8.675	D04:50103153..50103725
GhbHLH252	Gh_D05G0023	232	26247.71	4.511	D05:251448..252479
GhbHLH253	Gh_D05G0247	332	35960.85	7.286	D05:2218075..2220168
GhbHLH254	Gh_D05G0303	521	58267.8	7.776	D05:2616811..2619060
GhbHLH255	Gh_D05G0369	504	54159.72	4.927	D05:3076615..3078952
GhbHLH256	Gh_D05G0534	450	47392.04	6.437	D05:4345739..4348426
GhbHLH257	Gh_D05G0688	427	47124.57	8.399	D05:5571985..5574369
GhbHLH258	Gh_D05G0699	263	29796.03	5.843	D05:5705011..5706001
GhbHLH259	Gh_D05G0789	836	91579.68	5.744	D05:6570950..6575495+
GhbHLH260	Gh_D05G0858	322	36248.11	4.883	D05:7211762..7213050

GhbHLH261	Gh_D05G1480	275	30496.7	8.337	D05:13300430..13302890
GhbHLH262	Gh_D05G1534	165	18879.79	9.811	D05:13862402..13863142
GhbHLH263	Gh_D05G1594	466	50950.77	5.323	D05:14417895..14420205
GhbHLH264	Gh_D05G1601	253	27798.42	5.941	D05:14472842..14474291
GhbHLH265	Gh_D05G1827	434	48020.74	6.877	D05:16609882..16612997
GhbHLH266	Gh_D05G1949	265	29114.02	8.364	D05:17914447..17915244
GhbHLH267	Gh_D05G1958	333	35762.82	5.666	D05:18023807..18026868
GhbHLH268	Gh_D05G2013	376	41440.87	4.525	D05:18497859..18499302
GhbHLH269	Gh_D05G2112	230	25644.11	8.556	D05:19713013..19713791
GhbHLH270	Gh_D05G2140	337	37332.12	5.947	D05:20032948..20034373
GhbHLH271	Gh_D05G2531	478	54766.35	4.999	D05:25559407..25561779
GhbHLH272	Gh_D05G2532	356	38784.13	4.659	D05:25590480..25591550
GhbHLH273	Gh_D05G2928	93	10570.09	10.002	D05:34656477..34656917
GhbHLH274	Gh_D05G2954	327	36442.31	5.891	D05:35445686..35448213
GhbHLH275	Gh_D05G3213	600	65731.34	7.121	D05:50013992..50018061
GhbHLH276	Gh_D05G3503	385	42753.46	6.879	D05:57686751..57689739
GhbHLH277	Gh_D05G3774	447	50809.97	8.573	scaffold4036_D05:28977..31727
GhbHLH278	Gh_D05G3821	340	38689.82	4.657	scaffold4068_D05:21799..23094
GhbHLH279	Gh_D06G0034	342	37909.31	9.013	D06:384379..387769
GhbHLH280	Gh_D06G0152	204	22546.04	9.034	D06:1518698..1519312
GhbHLH281	Gh_D06G0217	414	45101.43	6.162	D06:2171958..2173889
GhbHLH282	Gh_D06G0273	406	44642.32	5.246	D06:3063306..3064895
GhbHLH283	Gh_D06G0622	352	38813.39	7.369	D06:10587297..10589805
GhbHLH284	Gh_D06G1019	477	51553.38	8.712	D06:21684341..21687399
GhbHLH285	Gh_D06G1312	932	102969.2	6.015	D06:39694173..39699533
GhbHLH286	Gh_D06G1384	178	20256.9	8.928	D06:43265338..43266130
GhbHLH287	Gh_D06G1739	426	47193.87	5.487	D06:56928371..56929905
GhbHLH288	Gh_D06G1740	429	47578.39	5.879	D06:56941722..56943265
GhbHLH289	Gh_D06G2316	331	37658.59	4.637	scaffold4092_D06:50019..51911
GhbHLH290	Gh_D07G0072	384	41975.97	8.564	D07:701022..705889
GhbHLH291	Gh_D07G0141	487	52054.32	4.943	D07:1514092..1516274
GhbHLH292	Gh_D07G0403	236	26187.7	9.239	D07:4381485..4382503
GhbHLH293	Gh_D07G0480	293	32831.17	8.451	D07:5314800..5317609
GhbHLH294	Gh_D07G0665	275	30922.68	6.394	D07:7783929..7785498
GhbHLH295	Gh_D07G0698	439	48399.64	9.054	D07:8313076..8316803
GhbHLH296	Gh_D07G0897	509	55551.36	7.458	D07:11648262..11651781
GhbHLH297	Gh_D07G1303	473	51142.27	8.908	D07:20731379..20733283
GhbHLH298	Gh_D07G1340	503	56223.55	5.18	D07:21512230..21513741
GhbHLH299	Gh_D07G1543	574	62750.81	6.677	D07:28526010..28530100
GhbHLH300	Gh_D07G1678	73	8734.32	9.784	D07:34867808..34868998
GhbHLH301	Gh_D07G1712	328	36424.86	5.373	D07:37300655..37303262
GhbHLH302	Gh_D07G1955	101	11956.05	9.671	D07:48794266..48796539
GhbHLH303	Gh_D07G2005	309	34519.71	7.134	D07:49697743..49699108
GhbHLH304	Gh_D07G2050	564	61452.1	6.148	D07:50348395..50353906

GhbHLH305	Gh_D07G2121	299	31678.99	7.62	D07:51484436..51486510
GhbHLH306	Gh_D07G2149	715	78948.31	6.205	D07:51879382..51885122-
GhbHLH307	Gh_D07G2183	94	10483.94	8.203	D07:52530221..52532305
GhbHLH308	Gh_D07G2352	477	52380.23	5.242	D07:55146576..55150280
GhbHLH309	Gh_D07G2371	521	58399.69	8.632	scaffold4168_D07:48710..52382
GhbHLH310	Gh_D07G2426	294	32486.15	6.222	scaffold4175_D07:68241..70341
GhbHLH311	Gh_D08G0317	339	38048.57	8.04	D08:3072505..3074712
GhbHLH312	Gh_D08G0565	255	28346.68	6.349	D08:6624734..6625763
GhbHLH313	Gh_D08G0646	733	80699.28	6.555	D08:8164362..8170213+
GhbHLH314	Gh_D08G0666	304	32016.26	6.361	D08:8844347..8848144
GhbHLH315	Gh_D08G1175	463	52001.99	6.477	D08:37340642..37342033
GhbHLH316	Gh_D08G1185	818	89511.41	5.854	D08:37894002..37897890
GhbHLH317	Gh_D08G1374	476	53054.8	6.577	D08:45029128..45030558
GhbHLH318	Gh_D08G1707	673	73447.76	5.435	D08:53180179..53182200
GhbHLH319	Gh_D08G1966	628	70240.7	6.14	D08:57987875..57990387
GhbHLH320	Gh_D08G1998	680	76178.19	4.985	D08:58452899..58458970
GhbHLH321	Gh_D08G2028	305	32118.31	5.964	D08:58796641..58799273
GhbHLH322	Gh_D08G2242	331	37416.08	8.15	D08:62031078..62032250
GhbHLH323	Gh_D09G0072	308	33955.76	6.816	D09:1892861..1895371
GhbHLH324	Gh_D09G0180	93	10568.66	9.548	D09:5731052..5731414
GhbHLH325	Gh_D09G0182	92	10412.86	8.205	D09:5840738..5841539
GhbHLH326	Gh_D09G0484	269	30489.28	7.89	D09:23469003..23470602
GhbHLH327	Gh_D09G0518	192	21349.85	8.17	D09:25042793..25044615
GhbHLH328	Gh_D09G0684	343	37643.7	7.094	D09:30679279..30682168
GhbHLH329	Gh_D09G0859	287	32370.5	4.78	D09:33613345..33614863
GhbHLH330	Gh_D09G0966	906	98612.16	6.033	D09:35229634..35235020-
GhbHLH331	Gh_D09G1014	386	43798.66	8.618	D09:36115545..36118611
GhbHLH332	Gh_D09G1180	374	40915.81	6.716	D09:38581585..38586684
GhbHLH333	Gh_D09G1183	376	41739.89	8.662	D09:38602543..38604331
GhbHLH334	Gh_D09G1679	283	32063.78	6.691	D09:44445546..44446898
GhbHLH335	Gh_D09G1697	229	25568.98	8.102	D09:44660361..44661317
GhbHLH336	Gh_D09G1771	160	18570.64	8.52	D09:45348479..45350509
GhbHLH337	Gh_D09G1895	662	72071.22	5.396	D09:46389958..46391946
GhbHLH338	Gh_D09G1900	295	33274.47	8.945	D09:46465260..46466430
GhbHLH339	Gh_D09G2157	254	29454.54	9.519	D09:48738427..48740921
GhbHLH340	Gh_D09G2368	514	56918.69	8.95	D09:50629085..50635358
GhbHLH341	Gh_D09G2423	442	49360.44	7.83	scaffold4319_D09:480..1328
GhbHLH342	Gh_D10G0179	213	24310.96	5.784	D10:1531450..1532789
GhbHLH343	Gh_D10G0181	352	38697.2	5.801	D10:1545803..1547754
GhbHLH344	Gh_D10G0240	186	20994.65	9.038	D10:2072903..2073620
GhbHLH345	Gh_D10G0295	258	28761.8	8.488	D10:2520482..2522543
GhbHLH346	Gh_D10G0600	539	59653.95	7.062	D10:6220089..6223365
GhbHLH347	Gh_D10G0646	328	35868.14	6.421	D10:6975064..6977764
GhbHLH348	Gh_D10G0984	270	30042.85	7.844	D10:13542063..13543027

GhbHLH349	Gh_D10G1033	223	25200.76	6.841	D10:15323310..15323981
GhbHLH350	Gh_D10G1206	149	16629.99	9.028	D10:20877966..20879780
GhbHLH351	Gh_D10G1288	333	35633.6	8.173	D10:23679940..23685259
GhbHLH352	Gh_D10G1331	511	55568.24	6.601	D10:25337714..25339790
GhbHLH353	Gh_D10G1411	409	46416.43	5.593	D10:29311931..29313337
GhbHLH354	Gh_D10G1533	213	24264.51	6.39	D10:39781448..39783664
GhbHLH355	Gh_D10G1580	73	8734.32	9.784	D10:43119671..43120853
GhbHLH356	Gh_D10G2011	637	72759.53	8.893	D10:55668749..55677545
GhbHLH357	Gh_D10G2228	267	30440.22	8.535	D10:59765890..59773516
GhbHLH358	Gh_D10G2406	238	26362.15	8.511	D10:63062175..63063754
GhbHLH359	Gh_D11G0045	214	24570.05	10.2	D11:418024..426231
GhbHLH360	Gh_D11G0213	624	69815.7	5.524	D11:1831806..1834212
GhbHLH361	Gh_D11G0306	285	30690.27	6.244	D11:2653527..2656326
GhbHLH362	Gh_D11G0327	92	10419.69	8.224	D11:2789505..2790528
GhbHLH363	Gh_D11G0328	89	9977.32	89	D11:2801737..2802231
GhbHLH364	Gh_D11G0332	217	23858.91	7.059	D11:2826526..2827793
GhbHLH365	Gh_D11G0373	550	60233.92	8.227	D11:3165096..3171289
GhbHLH366	Gh_D11G0411	333	37376.08	8.081	D11:3443954..3445723
GhbHLH367	Gh_D11G0415	555	61199.26	6.347	D11:3475705..3478151
GhbHLH368	Gh_D11G0530	302	34380.97	6.856	D11:4675489..4677195
GhbHLH369	Gh_D11G0543	210	24124.25	10.389	D11:4764584..4765780
GhbHLH370	Gh_D11G0675	394	42807.88	7.002	D11:5885188..5887914
GhbHLH371	Gh_D11G0826	496	54128.01	8.756	D11:7123324..7126574
GhbHLH372	Gh_D11G0862	116	13541.81	9.235	D11:7399364..7401556
GhbHLH373	Gh_D11G1032	616	68102.48	6.551	D11:9066667..9068517
GhbHLH374	Gh_D11G1055	495	55344.62	5.36	D11:9471747..9473234
GhbHLH375	Gh_D11G1063	385	43732.62	5.982	D11:9651093..9652904
GhbHLH376	Gh_D11G1107	558	61979.42	7.593	D11:10101819..10104002
GhbHLH377	Gh_D11G1266	346	39003.63	5.115	D11:12023554..12025768
GhbHLH378	Gh_D11G1273	655	74139.87	5.38	D11:12104370..12108609
GhbHLH379	Gh_D11G1319	303	31953.14	6.549	D11:12697173..12701013
GhbHLH380	Gh_D11G1337	504	55566.87	5.64	D11:12898369..12899883
GhbHLH381	Gh_D11G1395	549	60047.59	6.775	D11:13756704..13759939
GhbHLH382	Gh_D11G1449	538	58664.7	8.072	D11:14475427..14478136
GhbHLH383	Gh_D11G1773	304	33809.51	5.274	D11:19763597..19765414
GhbHLH384	Gh_D11G1935	267	29878.55	9.018	D11:24143297..24144100
GhbHLH385	Gh_D11G1945	394	43148.07	5.433	D11:24299237..24302094
GhbHLH386	Gh_D11G2073	362	40090.1	7.094	D11:29067150..29068519
GhbHLH387	Gh_D11G2074	329	37238.27	6.172	D11:29160438..29161804
GhbHLH388	Gh_D11G2082	349	39645.81	4.981	D11:29769005..29770314
GhbHLH389	Gh_D11G2383	80	9176.61	8.563	D11:46876530..46877042
GhbHLH390	Gh_D11G2556	327	34125.69	5.962	D11:52832641..52836509
GhbHLH391	Gh_D11G2701	314	34749.9	6.463	D11:56288953..56290074
GhbHLH392	Gh_D11G2839	705	76373.2	5.99	D11:58333435..58336628

GhbHLH393	Gh_D11G2890	237	26404.97	7.912	D11:58948074..59010257
GhbHLH394	Gh_D11G3081	256	29032.11	6.535	D11:62770013..62772513
GhbHLH395	Gh_D11G3162	309	33866.16	5.939	D11:64229182..64232704
GhbHLH396	Gh_D11G3340	233	26265.97	7.815	scaffold4488_D11:41302..43591
GhbHLH397	Gh_D11G3523	412	46994.4	6.539	scaffold4566_D11:22094..23980
GhbHLH398	Gh_D12G0259	423	46652.68	5.874	D12:3511001..3513410
GhbHLH399	Gh_D12G0328	602	68188.52	5.232	D12:4670664..4673082
GhbHLH400	Gh_D12G0475	262	28892.25	7.454	D12:7969982..7973414
GhbHLH401	Gh_D12G0497	246	27892.66	6.878	D12:8532227..8534106
GhbHLH402	Gh_D12G0607	358	39607.15	6.469	D12:11463442..11465086
GhbHLH403	Gh_D12G0670	241	26816.21	7.278	D12:14002245..14004272
GhbHLH404	Gh_D12G1160	487	54431.14	5.527	D12:38662690..38664153
GhbHLH405	Gh_D12G1398	212	24875.51	8.512	D12:43127063..43127870
GhbHLH406	Gh_D12G1443	470	53475.44	8.383	D12:44285423..44287901
GhbHLH407	Gh_D12G1583	297	32849.26	6.389	D12:47029047..47031500
GhbHLH408	Gh_D12G1645	92	10354.74	9.584	D12:47994524..47995011
GhbHLH409	Gh_D12G1688	345	38154.14	5.952	D12:48728273..48730066
GhbHLH410	Gh_D12G1740	87	9678.92	5.645	D12:49644596..49645123
GhbHLH411	Gh_D12G1906	337	37398.07	7.594	D12:51904842..51906772
GhbHLH412	Gh_D12G2069	331	37558.28	8.443	D12:53727335..53729500
GhbHLH413	Gh_D12G2074	646	70962.24	5.646	D12:53800306..53802246
GhbHLH414	Gh_D12G2351	475	53045.76	6.626	D12:56642751..56644178
GhbHLH415	Gh_D12G2505	205	22685.65	5.534	D12:57984938..57985784
GhbHLH416	Gh_D12G2506	300	34042.32	9.188	D12:57990142..57991983
GhbHLH417	Gh_D12G2689	329	36063.12	5.104	scaffold4579_D12:24745..26275
GhbHLH418	Gh_D13G0207	262	30128	8.961	D13:2060162..2063581
GhbHLH419	Gh_D13G0330	428	47139.4	6.258	D13:3315222..3316873
GhbHLH420	Gh_D13G0346	363	40909.97	5.708	D13:3493999..3496526
GhbHLH421	Gh_D13G0406	266	29433.13	6.947	D13:4497729..4500458
GhbHLH422	Gh_D13G0711	380	41558.35	8.967	D13:11030215..11032176
GhbHLH423	Gh_D13G0759	89	9895.18	6.593	D13:12277553..12278227
GhbHLH424	Gh_D13G0896	327	36384.14	6.565	D13:18147693..18150283
GhbHLH425	Gh_D13G0902	554	58954.25	5.864	D13:18250550..18253184
GhbHLH426	Gh_D13G1180	199	22574.07	11.439	D13:35330946..35331545
GhbHLH427	Gh_D13G1248	269	29370.78	7.162	D13:38141857..38145627
GhbHLH428	Gh_D13G1496	238	26371.81	6.629	D13:46586468..46589066
GhbHLH429	Gh_D13G1856	287	32478.86	7.88	D13:53691959..53692983
GhbHLH430	Gh_D13G2183	245	27805.72	5.105	D13:57964607..57970742
GhbHLH431	Gh_D13G2548	249	26648.69	6.593	scaffold4714_D13:9236..10279
GhbHLH432	Gh_Sca004901G02	267	29991.67	9.018	scaffold4901:36005..36808
GhbHLH433	Gh_Sca008309G01	149	16628.6	8.161	scaffold8309:4..1911
GhbHLH434	Gh_Sca008688G01	254	28496.43	6.344	scaffold8688:961..2225
GhbHLH435	Gh_Sca014319G01	129	14962.44	7.039	scaffold14319:17..1117
GhbHLH436	Gh_Sca024466G01	99	11424.11	9.687	scaffold24466:1126..1422

GhbHLH437	Gh_Sca038875G01	312	34584.2	6.625	scaffold38875:40..978

Length: the length of the encoding protein (aa, amino acid); pI: the theoretical isoelectric point of the encoding protein; Mw: the theoretical molecular weight of the encoding protein.

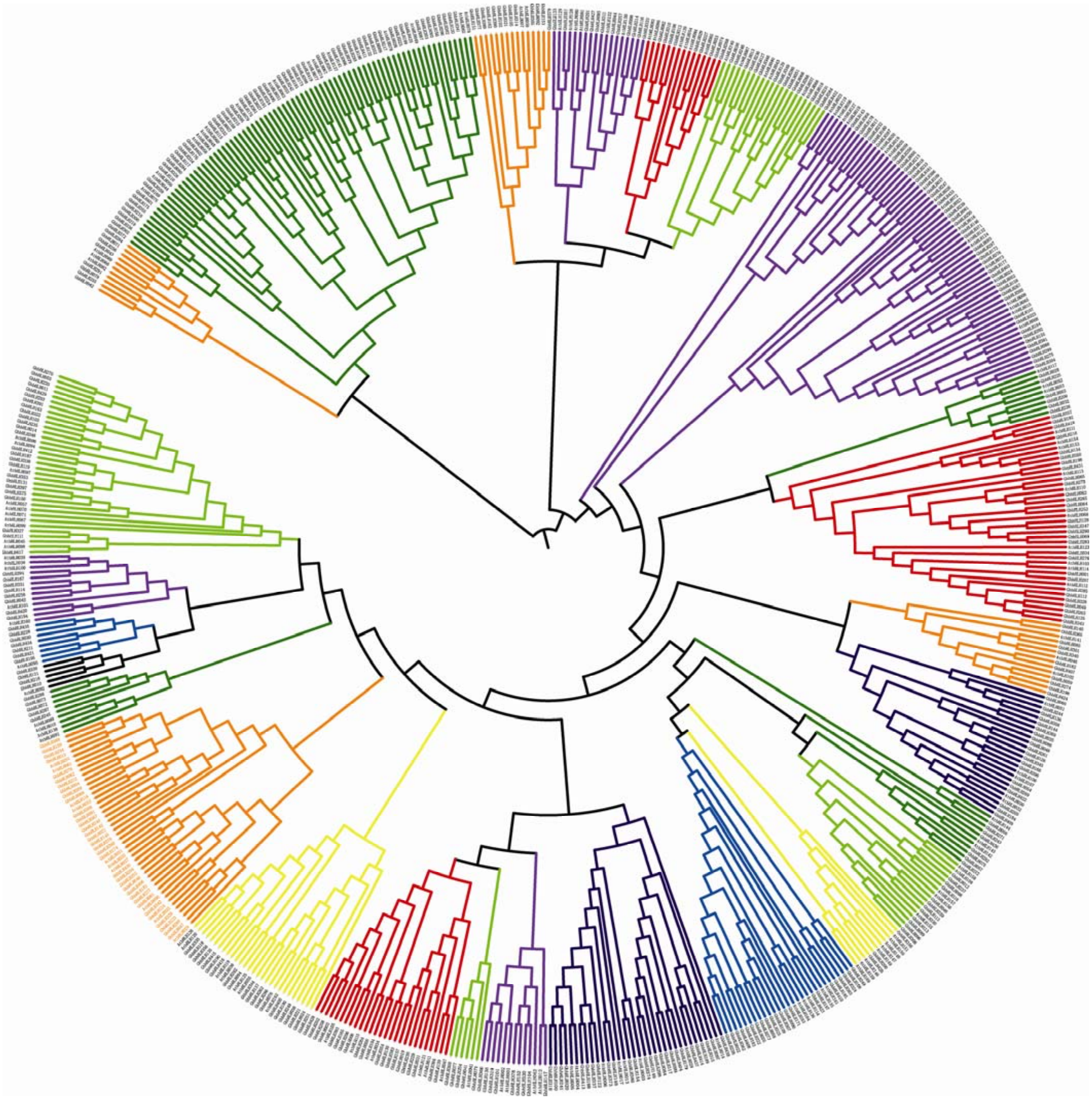

Figure S1. Phylogenetic relationship of cotton (*G*ossypium *hirsutum*) bHLH transcription factors with those of Arabidopsis and rice.** MEGA 6.0 software was employed to construct an unrooted phylogenetic tree based on alignments using the NeighborJoining (NJ) method with the following parameters: the number of differences model, pairwise deletion and 1,000 bootstraps.

Table S2. Phylogenetic classification and known biological functions of bHLH/HLH proteins from Arabidopsis and cotton (*Gossypium hirsutum*).

Subfamily	Arabidopsis bHLH/HLHs	cotton bHLH/HLHs		Arabidopsis mutants and gene functions
**S1**	AtbHLH021	GhbHLH004	GhbHLH212	ice1, Reduction in plant chilling and freezing tolerance (AtbHLH033)
	AtbHLH022	GhbHLH015	GhbHLH234	fit1, Defects in Fe uptake (AtbHLH029)
	AtbHLH027	GhbHLH021	GhbHLH240	ICE2, Cold acclimation (AtbHLH035)
	AtbHLH029	GhbHLH040	GhbHLH252	dyt1, Defects in the tapetum at anther stage 4 and later (AtbHLH022)
	AtbHLH033	GhbHLH047	GhbHLH260	ams, Sporophytic male sterile (AtbHLH021)
	AtbHLH035	GhbHLH061	GhbHLH277	BHLH093, NFL, NO FLOWERING IN SHORT DAY. nfl, short stature,
	AtbHLH061	GhbHLH062	GhbHLH278	curly and dark green leaves and reduced fertility. Late flowering under short days.
	AtbHLH090	GhbHLH068	GhbHLH289	
	AtbHLH093	GhbHLH091	GhbHLH308	
	AtbHLH116	GhbHLH123	GhbHLH329	
		GhbHLH124	GhbHLH342	
		GhbHLH142	GhbHLH367	
		GhbHLH145	GhbHLH370	
		GhbHLH159	GhbHLH388	
		GhbHLH174	GhbHLH399	
		GhbHLH181	GhbHLH406	
		GhbHLH204	GhbHLH430	
**S2**	AtbHLH003	GhbHLH020	GhbHLH298	MYC4, JAZ-interacting activator of JA-responses (AtbHLH004)
	AtbHLH004	GhbHLH084	GhbHLH315	MYC3, JAZ-interacting activator of JA-responses (AtbHLH005)
	AtbHLH005	GhbHLH097	GhbHLH317	MYC2, JAZ-interacting activator of JA-responses (AtbHLH006)
	AtbHLH006	GhbHLH099	GhbHLH318	
	AtbHLH013	GhbHLH100	GhbHLH337	bhlh3 bhlh13 bhlh14 bhlh17, increased JA sensitivity (AtbHLH003,
	AtbHLH014	GhbHLH122	GhbHLH373	AtbHLH013, AtbHLH014 and AtbHLH017)
	AtbHLH017	GhbHLH148	GhbHLH374	ataib, Weak insensitivity to ABA (AtbHLH013)
	AtbHLH028	GhbHLH149	GhbHLH380	atnig1-1, Reduction in resistance to salt stress (AtbHLH017)

		GhbHLH154	GhbHLH404	
		GhbHLH179	GhbHLH413	
		GhbHLH188	GhbHLH414	
		GhbHLH189	GhbHLH437	
		GhbHLH241		
**S3**	AtbHLH018	GhbHLH005	GhbHLH213	ER development; response to endophytic fungus (AtbHLH020)
	AtbHLH019	GhbHLH016	GhbHLH232	
	AtbHLH020	GhbHLH017	GhbHLH233	
	AtbHLH025	GhbHLH039	GhbHLH250	
		GhbHLH143	GhbHLH368	
		GhbHLH160	GhbHLH386	
		GhbHLH161	GhbHLH387	
		GhbHLH178		
**S4**	AtbHLH011	GhbHLH002	GhbHLH206	
	AtbHLH034	GhbHLH008	GhbHLH208	PYE, Regulator of response to iron deficiency (AtbHLH047)
	AtbHLH047	GhbHLH019	GhbHLH229	
	AtbHLH104	GhbHLH031	GhbHLH230	ILR3, conjugated IAA metabolism (AtbHLH105)
	AtbHLH105	GhbHLH050	GhbHLH264	
	AtbHLH115	GhbHLH130	GhbHLH354	
	AtbHLH121	GhbHLH139	GhbHLH364	
		GhbHLH157	GhbHLH383	
		GhbHLH165	GhbHLH393	
		GhbHLH202	GhbHLH428	
**S5**	AtbHLH001	GhbHLH101	GhbHLH319	gl3-1, trichomes unbranched and reduced in number (AtbHLH001)
	AtbHLH002	GhbHLH104	GhbHLH320	egl3-1, reduced trichomes, anthocyanin, and seed coat mucilage and
	AtbHLH012	GhbHLH138	GhbHLH360	abnormally patterned stomata. (AtbHLH002)
	AtbHLH042	GhbHLH147	GhbHLH378	myc1-1, Increased ectopic root-hair cells (AtbHLH012)
		GhbHLH152		
**S7a**	AtbHLH041	GhbHLH041	GhbHLH254	
		GhbHLH070	GhbHLH309	
		GhbHLH077		
**S7b**	AtbHLH092	GhbHLH010	GhbHLH218	
		GhbHLH121	GhbHLH339	

**S9**	AtbHLH010<br>AtbHLH089<br>AtbHLH091<br>AtbHLH138	GhbHLH072<br>GhbHLH073<br>GhbHLH245<br>GhbHLH287	GhbHLH288	
**S10**	AtbHLH045<br>AtbHLH057<br>AtbHLH067<br>AtbHLH070<br>AtbHLH071<br>AtbHLH094<br>AtbHLH096<br>AtbHLH097<br>AtbHLH098<br>AtbHLH099	GhbHLH011<br>GhbHLH014<br>GhbHLH055<br>GhbHLH103<br>GhbHLH111<br>GhbHLH119<br>GhbHLH131<br>GhbHLH150<br>GhbHLH163<br>GhbHLH187<br>GhbHLH203<br>GhbHLH220<br>GhbHLH235	GhbHLH270<br>GhbHLH322<br>GhbHLH327<br>GhbHLH338<br>GhbHLH348<br>GhbHLH353<br>GhbHLH375<br>GhbHLH391<br>GhbHLH397<br>GhbHLH412<br>GhbHLH417<br>GhbHLH429	SPCH, necessary and sufficient for the asymmetric divisions that establish the stomatal lineage (AtbHLH099)<br>mtp1-1, Enhanced sensitivity to elevated Zn (AtbHLH071)<br>Mute, Complete absence of stomata (AtbHLH045)<br>fama-1, Failed differentiation of guard cells (AtbHLH098)
**S11**	AtbHLH095	GhbHLH030<br>GhbHLH195<br>GhbHLH211<br>GhbHLH228	GhbHLH421<br>GhbHLH434<br>GhbHLH435	zou, retarded embryo growth
**S12a**	AtbHLH038<br>AtbHLH039<br>AtbHLH100<br>AtbHLH101<br>AtbHLH160	GhbHLH045<br>GhbHLH114<br>GhbHLH167<br>GhbHLH194<br>GhbHLH258	GhbHLH331<br>GhbHLH394<br>GhbHLH420	ORG2 (AtbHLH038), ORG3 (AtbHLH039), response to iron ion, response to SA
**S12b**	AtbHLH036<br>AtbHLH055<br>AtbHLH118<br>AtbHLH120<br>AtbHLH125<br>AtbHLH126<br>AtbHLH162	GhbHLH024<br>GhbHLH038<br>GhbHLH076<br>GhbHLH094<br>GhbHLH118<br>GhbHLH135<br>GhbHLH137	GhbHLH251<br>GhbHLH262<br>GhbHLH286<br>GhbHLH292<br>GhbHLH335<br>GhbHLH344<br>GhbHLH356	

		GhbHLH180<br>GhbHLH190<br>GhbHLH221<br>GhbHLH248	GhbHLH405<br>GhbHLH415<br>GhbHLH416	
**S13**	AtbHLH030<br>AtbHLH032<br>AtbHLH051<br>AtbHLH106<br>AtbHLH107<br>AtbHLH131	GhbHLH035<br>GhbHLH048<br>GhbHLH054<br>GhbHLH060<br>GhbHLH086<br>GhbHLH126<br>GhbHLH136<br>GhbHLH144<br>GhbHLH166<br>GhbHLH184	GhbHLH205<br>GhbHLH244<br>GhbHLH261<br>GhbHLH269<br>GhbHLH345<br>GhbHLH358<br>GhbHLH369<br>GhbHLH396<br>GhbHLH409<br>GhbHLH422	ATbHLH032, expression of PPCK is elevated under Pi starvation. Under high levels of Pi, root hair contained significantly more total Pi and more anthocyanin than the wild-type. DFR expression in Pi-sufficient conditions was substantially increased.
**S14**	AtbHLH046<br>AtbHLH102<br>AtbHLH141	GhbHLH059<br>GhbHLH085<br>GhbHLH140<br>GhbHLH182<br>GhbHLH198<br>GhbHLH274	GhbHLH301<br>GhbHLH340<br>GhbHLH365<br>GhbHLH407<br>GhbHLH424	BIM (BES1-INTERACTING MYC-LIKE)1 (AtbHLH046), synergistically interacts with BES1 to bind to E box sequences (CANNTG). Positively modulates the shade avoidance syndrome in Arabidopsis seedlings.<br>BIM3 (AtbHLH141) and BIM2 (AtbHLH102), PAR1 (PHYTOCHROME RAPIDLY REGULATED 1)-interacting proteins that positively modulates the shade avoidance syndrome in Arabidopsis seedlings.
**S15**	AtbHLH068<br>AtbHLH103<br>AtbHLH110<br>AtbHLH111<br>AtbHLH112<br>AtbHLH113<br>AtbHLH114<br>AtbHLH123<br>AtbHLH133<br>AtbHLH153<br>AtbHLH154	GhbHLH001<br>GhbHLH034<br>GhbHLH049<br>GhbHLH063<br>GhbHLH064<br>GhbHLH065<br>GhbHLH069<br>GhbHLH112<br>GhbHLH125<br>GhbHLH128<br>GhbHLH134<br>GhbHLH192<br>GhbHLH196<br>GhbHLH207	GhbHLH253<br>GhbHLH263<br>GhbHLH265<br>GhbHLH276<br>GhbHLH279<br>GhbHLH283<br>GhbHLH285<br>GhbHLH290<br>GhbHLH328<br>GhbHLH343<br>GhbHLH347<br>GhbHLH350<br>GhbHLH357<br>GhbHLH418	ERP (AtbHLH154) :Response to ethylene and gibberellin stimulus.

		GhbHLH216	GhbHLH431	
**S16**	AtbHLH134(PRE2) AtbHLH135(PRE3) AtbHLH136(PRE1) AtbHLH161(PRE4) AtbHLH163(PRE6) AtbHLH164(PRE5)	GhbHLH022 GhbHLH025 GhbHLH058 GhbHLH090 GhbHLH108 GhbHLH109 GhbHLH171 GhbHLH183 GhbHLH185 GhbHLH197 GhbHLH222	GhbHLH238 GhbHLH249 GhbHLH273 GhbHLH307 GhbHLH324 GhbHLH325 GhbHLH362 GhbHLH363 GhbHLH408 GhbHLH410 GhbHLH423	AtbHLH135, ATBS1(activation-tagged bri1(brassinosteroid-insensitive 1)-suppressor 1), TMO7, target of monopteros 7, PRE3, paclobutrazol resistance 3. BNQ1(AtbHLH136), BNQ2 (AtbHLH134, PRE2) and BNQ3(AtbHLH161) directly and negatively regulated by AP3 and PI in petals, required for appropriate regulation of flowering time. AtbHLH136, PRE1 and IBH1 form a pair of antagonistic HLH/bHLH transcription factors that function downstream of BZR1 to mediate brassinosteroid regulation of cell elongation. bnq3, sepals and carpels are pale yellow or white,while the inflorescence stems and siliques are purple. Floral organs are smaller than WT. Flowers, cauline leaves, stems, and siliques have a decreased amount of chlorophyll as compared to WT. AtbHLH163 (KDR, PRE6), involved in blue/far-red light signaling. Physically interacts with HFR1 and negatively regulates its activity. Auxin response
**S17**	AtbHLH142 AtbHLH143 AtbHLH144 AtbHLH145	GhbHLH056 GhbHLH057 GhbHLH075 GhbHLH243	GhbHLH271 GhbHLH272 GhbHLH282 GhbHLH436	sac51-d, Upregulation of SAC51 [suppressor of acaulis 51] (AtbHLH142) reverses the dwarf phenotype caused by a loss-of-function in ACL5 gene encoding spermine synthase.
**S18**	AtbHLH146 AtbHLH147 AtbHLH148 AtbHLH149 AtbHLH150 AtbHLH151 AtbHLH159 AtbHLH165 AtbHLH166 AtbHLH167 AtbHLH168 AtbHLH169 AtbHLH170	GhbHLH200 GhbHLH426		AtbHLH158, ILI1binding bHLH 1 (IBH1) . AtbHLH150, AtBS1(activation-tagged BRI1 suppressor 1)-interacting factor 1 (AIF1). AtbHLH148, AIF2. AtbHLH147, AIF3. AtbHLH149, AIF4. AtbHLH167, PIR1. AtbHLH159, PIR2. AtbHLH168, PIR3. AtbHLH165, PHYTOCHROME RAPIDLY REGULATED1 (PAR1), and AtbHLH166 (PAR2), Up regulated after simulated shade perception. Acts in the nucleus to control plant development and as a negative regulator of shade avoidance response. Functions as transcriptional repressor of auxin-responsive genes SAUR15 and SAUR68.
**S23**	AtbHLH155 AtbHLH156	GhbHLH012 GhbHLH046	GhbHLH237 GhbHLH259	AtbHLH151, UPBEAT1 (UPB1), regulates the expression of a set of peroxidases that modulate the balance of reactive oxygen species (ROS) between the zones of cell proliferation and the zone

	AtbHLH157<br>AtbHLH158<br>AtbHLH108	GhbHLH089<br>GhbHLH096<br>GhbHLH098<br>GhbHLH113	GhbHLH306<br>GhbHLH313<br>GhbHLH316<br>GhbHLH330	of cell elongation where differentiation begins. Disruption of UPB1 activity alters this ROS balance, leading to a delay in the onset of differentiation. Root development. upb1-1, longer root than the wild type; significant increase in cortex cell number indicating enlargement of the meristem.<br>AtbHLH155, conserved peptide upstream open reading frame 7 (CPUORF7).<br>AtbHLH156 (LHW), promotes the production of stele cells in root meristems and is required to establish and maintain the normal vascular cell number and pattern in primary and lateral roots. lhw-1, reduced root vascular population. Roots lose bilateral symmetry and are monarch instead of diarch.
**S24**	AtbHLH008<br>AtbHLH009<br>AtbHLH015<br>AtbHLH016<br>AtbHLH023<br>AtbHLH024<br>AtbHLH026<br>AtbHLH056<br>AtbHLH065<br>AtbHLH072<br>AtbHLH073<br>AtbHLH109<br>AtbHLH119<br>AtbHLH124<br>AtbHLH127<br>AtbHLH132	GhbHLH023<br>GhbHLH037<br>GhbHLH052<br>GhbHLH081<br>GhbHLH083<br>GhbHLH088<br>GhbHLH107<br>GhbHLH146<br>GhbHLH155<br>GhbHLH158<br>GhbHLH164<br>GhbHLH172<br>GhbHLH177<br>GhbHLH239<br>GhbHLH247<br>GhbHLH267<br>GhbHLH275<br>GhbHLH295<br>GhbHLH297<br>GhbHLH299<br>GhbHLH304<br>GhbHLH323<br>GhbHLH371<br>GhbHLH376<br>GhbHLH381		AtbHLH015, PIF1, a key negative regulator of phytochrome-mediated seed germination and acts by inhibiting chlorophyll biosynthesis, light-mediated suppression of hypocotyl elongation and far-red light-mediated suppression of seed germination, and promoting negative gravitropism in hypocotyls.<br>AtbHLH065, PIL6, physically associated with APRR1/TOC1 and is a member of PIF3 transcription factor family. Involved in shade avoidance. Functions as negative regulator of PhyB.<br>AtbHLH072, PIF7, interacts specifically with the far-red light-absorbing Pfr form of phyB through a conserved domain called the active phyB binding motif. Upon light exposure, PIF7 rapidly migrates to intranuclear speckles, where it colocalizes with phyB. Role as negative regulator of phyB-mediated seedling deetiolation.<br>AtbHLH016, unfertilized embryo sac 10 (UNE10);<br>AtbHLH124 (PIL1), AtbHLH132(PIL2), physically associated with APRR1/TOC1 and are members of PIF3 transcription factor family. pil1, seedlings exhibit a significant shift (~6 h) in the phase of the circadian rhythm of hypocotyl elongation responses to low R/FR.<br>AtbHLH008 (PIF3), interacting with photoreceptors phyA and phyB. Forms a ternary complex in vitro with G-box element of the promoters of LHY, CCA1. Acts as a negative regulator of phyB signalling.<br>AtbHLH024 (SPT), spt-2, gynoecium flattened medially at apex, sometimes unfused at apex, silique shorter, flattened laterally at apex, style and stigma reduced, transmitting tract absent, reduced seed set.<br>AtbHLH073, ALCATRAZ(ALC), involved in fruit dehiscence. Mutant siliques fail to dehisce.<br>AtbHLH026, HFR1 (long hypocotyl in far-red) involved in phytochrome signaling. Mutants exhibit a long-hypocotyl phenotype only under far-red light but not under red light and are defective in other phytochrome A-related responses. Mutants also show blue light response

		GhbHLH385<br>GhbHLH392<br>GhbHLH403<br>GhbHLH433		defects. HFR1 interacts with COP1, co-localizes to the nuclear specks and is ubiquinated by COP1. hfr1, Long hypocotyl in far-red light
**S25**	AtbHLH031<br>AtbHLH044<br>AtbHLH048<br>AtbHLH049(ACE1)<br>AtbHLH050<br>AtbHLH058<br>AtbHLH060<br>AtbHLH062<br>AtbHLH063<br>AtbHLH064<br>AtbHLH074(ACE2)<br>AtbHLH075<br>AtbHLH076<br>AtbHLH077(ACE3)<br>AtbHLH078<br>AtbHLH079<br>AtbHLH137	GhbHLH007<br>GhbHLH009<br>GhbHLH026<br>GhbHLH027<br>GhbHLH033<br>GhbHLH067<br>GhbHLH079<br>GhbHLH080<br>GhbHLH087<br>GhbHLH093<br>GhbHLH110<br>GhbHLH115<br>GhbHLH117<br>GhbHLH120<br>GhbHLH132<br>GhbHLH141<br>GhbHLH156<br>GhbHLH168<br>GhbHLH169<br>GhbHLH170<br>GhbHLH173<br>GhbHLH175<br>GhbHLH176<br>GhbHLH186<br>GhbHLH191<br>GhbHLH193<br>GhbHLH199<br>GhbHLH210<br>GhbHLH215<br>GhbHLH217	GhbHLH223<br>GhbHLH224<br>GhbHLH242<br>GhbHLH281<br>GhbHLH293<br>GhbHLH294<br>GhbHLH296<br>GhbHLH300<br>GhbHLH302<br>GhbHLH303<br>GhbHLH326<br>GhbHLH332<br>GhbHLH334<br>GhbHLH336<br>GhbHLH341<br>GhbHLH352<br>GhbHLH355<br>GhbHLH359<br>GhbHLH361<br>GhbHLH366<br>GhbHLH372<br>GhbHLH382<br>GhbHLH395<br>GhbHLH398<br>GhbHLH400<br>GhbHLH401<br>GhbHLH402<br>GhbHLH411<br>GhbHLH419<br>GhbHLH425	AtbHLH063, CIB1, interacts with CRY2 (cryptochrome 2) in a blue light-specific manner in yeast and Arabidopsis cells, and it acts together with additional CIB1-related proteins to promote CRY2-dependent floral initiation. CIB1 positively regulates FT expression. cib1cib5 double mutant showed a mild but statistically significant delay of flowering under a photoperiodic inductive condition for 4 days, and removed back to short-day to continue grow until flowering.<br>AtbHLH058, a brassinosteroid signaling component BEE2 (BR-ENHANCED EXPRESSION 2). Positively modulates the shade avoidance syndrome in Arabidopsis seedlings.<br>AtbHLH064, HBI1(homolog of BEE2 interacting with ibh 1)<br>AtbHLH044, AtbHLH050, brassinosteroid signaling components BEE1 and BEE3. Positively modulates the shade avoidance syndrome in Arabidopsis seedlings.<br>AtbHLH075, CESTA, a positive regulator of brassinosteroid biosynthesis. AtbHLH076, CIB5 (cryptochrome-interacting basic-helix-loop-helix), interacts with CRY2 and forms heterodimer with CIB1 in vitro. Regulates flowering time redundantly with CIB1.<br>AtbHLH031, BIGPETAL, BPE, involved in the control of petal size. BPE is expressed via two mRNAs derived from an alternative splicing event. The BPEub (AT1G59640.1) transcript is expressed ubiquitously, whereas the BPEp (AT1G59640.2) transcript is preferentially expressed in petals. Plants that lack the petal-expressed variant BPEp have larger petals as a result of increased cell size. BPEp is positively regulated downstream of APETALA3, PISTILLATA, APETALA1 and PISTILLATA3 and is negatively regulated downstream of AGAMOUS. bigpetal-1, •Larger petal size as a result of increased petal cell size.

		GhbHLH219		
**S26**	AtbHLH007<br>AtbHLH059<br>AtbHLH066<br>AtbHLH069<br>AtbHLH082	GhbHLH042<br>GhbHLH043<br>GhbHLH071<br>GhbHLH078<br>GhbHLH092<br>GhbHLH102<br>GhbHLH105<br>GhbHLH151<br>GhbHLH153<br>GhbHLH162<br>GhbHLH255	GhbHLH256<br>GhbHLH284<br>GhbHLH291<br>GhbHLH305<br>GhbHLH314<br>GhbHLH321<br>GhbHLH377<br>GhbHLH379<br>GhbHLH389<br>GhbHLH390	AtbHLH066 (AtLRL1), AtbHLH069 (AtLRL2), and AtbHLH082 (AtLRL3), Arabidopsis homologs of the Lotus japonicus ROOTHAIRLESS1 (LjRHL1) gene.<br>AbHLH059, unfertilized embryo sac 12 (UNE12).
**S27**	AtbHLH080<br>AtbHLH081<br>AtbHLH122<br>AtbHLH128<br>AtbHLH129<br>AtbHLH130	GhbHLH006<br>GhbHLH044<br>GhbHLH095<br>GhbHLH116<br>GhbHLH133<br>GhbHLH201	GhbHLH214<br>GhbHLH257<br>GhbHLH311<br>GhbHLH333<br>GhbHLH351<br>GhbHLH427	AtbHLH081, FBH2 (flowering bhlh 2).<br>AtbHLH122, FBH3(flowering bhlh 3), AKS1(aba-responsive kinase substrate 1), involved in photoperiodism flowering. CFLAP1, participating in synergistic regulation of AtCFL1-mediated cuticle development.<br>AtbHLH130, FBH4(flowering bhlh 3); AKS3(aba-responsive kinase substrate 3).
**S28**	AtbHLH054<br>AtbHLH083<br>AtbHLH084<br>AtbHLH085<br>AtbHLH086<br>AtbHLH139	GhbHLH029<br>GhbHLH053<br>GhbHLH074<br>GhbHLH082<br>GhbHLH106<br>GhbHLH227	GhbHLH268<br>GhbHLH310<br>GhbHLH312	AtbHLH84, RSL3.<br>AtbHLH085, RSL2(root hair defective 6-like 2), expressed concurrently with RSL4 and its expression was controlled by RHD6 and RSL1. Required for root-hair growth.<br>AtbHLH054, RSL4, sufficient to promote postmitotic cell growth in root-hair cells, is a direct transcriptional target of RHD6.<br>AtbHLH083, ROOT HAIR DEFECTIVE 6 (RHD6). rhd6-1, altered root hair initiation; reduced number of root hairs; overall basal shift in the site of root hair emergency; relatively high frequency of epidermal cells with multiple root hairs; abnormal root hair phenotype is suppressed by ethylene or auxin..<br>AtbHLH86, RHD SIX-LIKE 1 (RSL1).
**S30**	AtbHLH052<br>AtbHLH053<br>AtbHLH117	GhbHLH003<br>GhbHLH028<br>GhbHLH032	GhbHLH209<br>GhbHLH225<br>GhbHLH226	
**S31**	AtbHLH037<br>AtbHLH040	GhbHLH013<br>GhbHLH018	GhbHLH236<br>GhbHLH246	AtbHLH88, HECATE 1 (HEC1), AtbHLH37 (HEC2), AtbHLH43, HEC3), involved in ovary septum development, transmitting tissue development, carpel formation. hec1 shows no alteration

	AtbHLH043<br>AtbHLH087<br>AtbHLH088<br>AtbHLH140	GhbHLH036<br>GhbHLH051<br>GhbHLH066<br>GhbHLH127<br>GhbHLH129<br>GhbHLH231	GhbHLH266<br>GhbHLH280<br>GhbHLH346<br>GhbHLH349<br>GhbHLH384<br>GhbHLH432	in fruit phenotype. hec3 transmitting tract was smaller in size in both the septum and the style, mutant carpels showed significantly fewer pollen tubes and pollination events, particularly in the basal half of the gynoecium.<br>AtbHLH040, INDEHISCENT (IND).

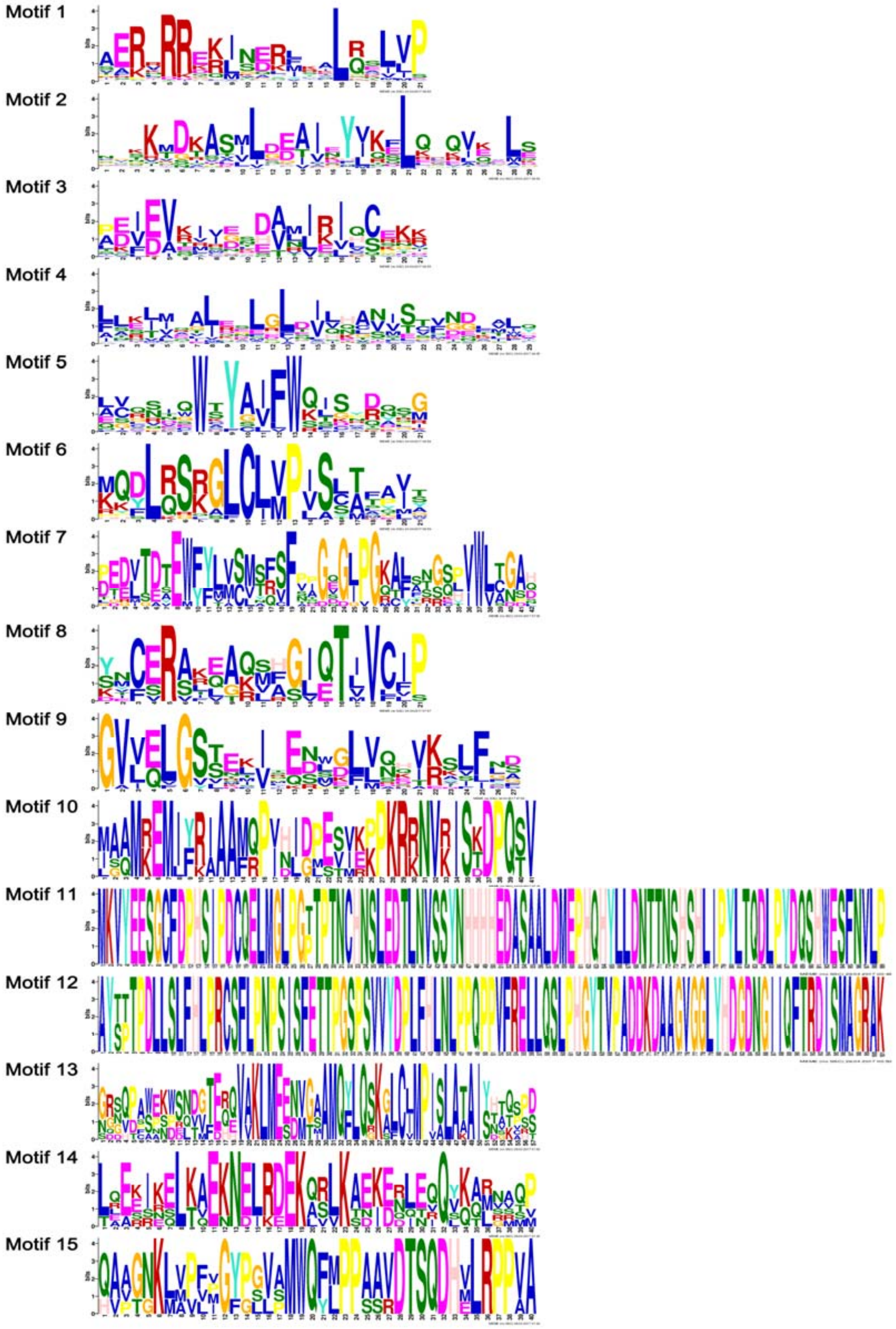

Figure S2. Conserved motifs of GhbHLH/HLH proteins. The motifs were identified using MEME program, and each motif is shown with the amino acid residue compositions.

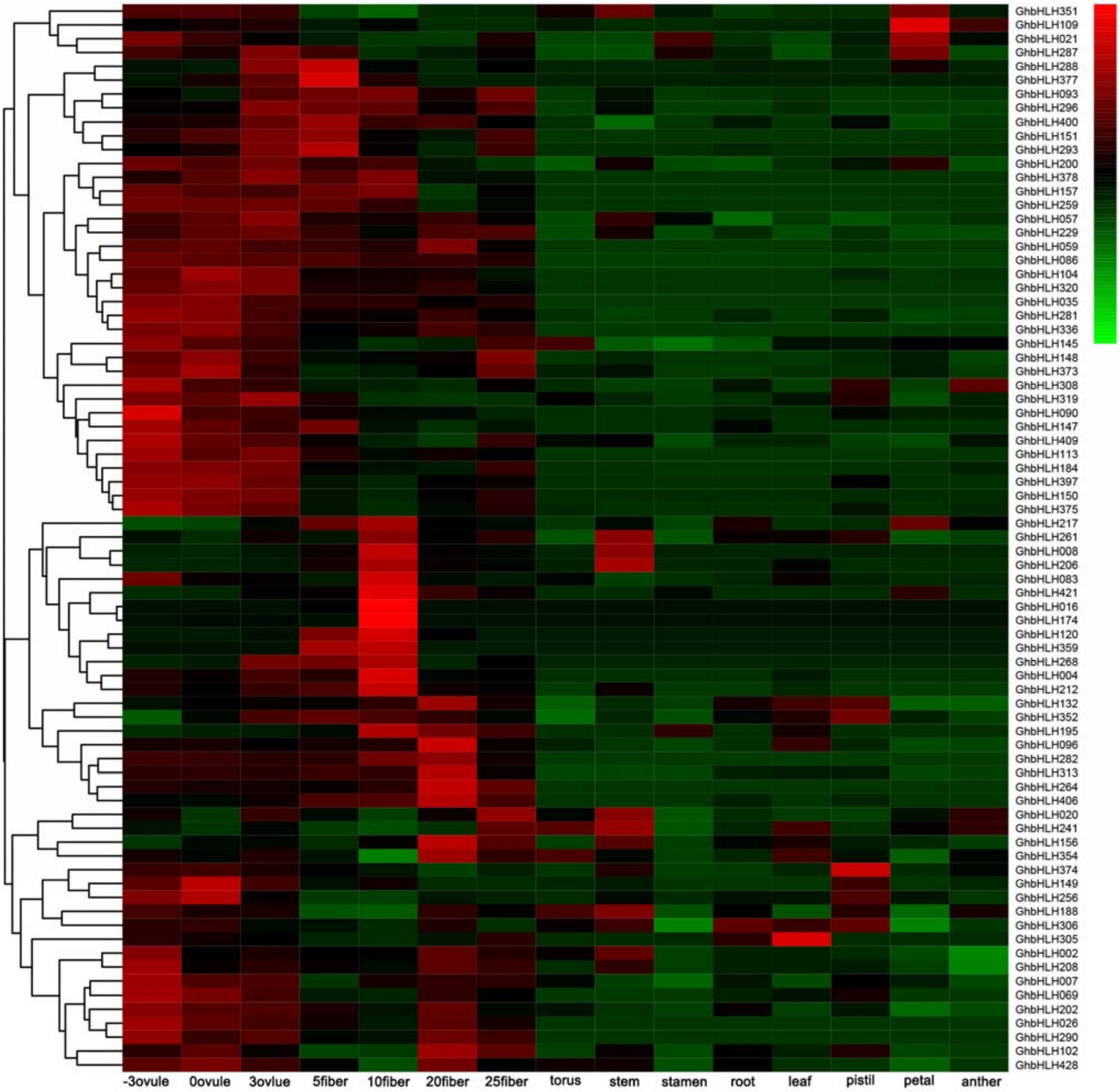

Figure S3. Heatmap representation for expression profiles of cotton (*G. hirsutum*) *bHLH/HLH* genes in cotton tissues. The different organs/tissues of cotton include ovules (–3, 0 and 3 DPA ovules), fibers (5, 10, 20 and 25 DPA fibers), torus, stems, stamens, roots, leaves, pistils, petals and anthers. Heatmaps for gene expression patterns was generated by the online program Omicsharea (http://www.omicshare.com/tools/Home/Soft/heatmap). –3ovule–3ovule, –3 – 3 DPA ovules; 5fiber–25fiber, 5 – 25 DPA fibers. DPA, day post anthesis.

Table S3. Primers used in this study.

Gene	Primers
GhbHLH001-F	GCACAACCTAGCTTCATGCCA
GhbHLH001-R	GGAGGACATGGTCCTGTGAA
GhbHLH002-F	GCTCTGAGGATCTGGAACAG
GhbHLH002-R	AACATGCTATTTCTTGCTGA
GhbHLH004-F	CACATCAGAAAGTGGATCTAC
GhbHLH004-R	CCAAATAGGCTGCTTAAGTGT
GhbHLH008-F	CGCGACGAGAAGCAGAGGCT
GhbHLH008-R	AAGAACGTGATCCTGTGATG
GhbHLH016-F	CCTCATTCGAATCCATTGTGAA
GhbHLH016-R	CTTTCTGAGCAATTATGGTG
GhbHLH020-F	GCTACAGAAGGTGGATTCAAT
GhbHLH020-R	AGTCGTTAACGACGGAAACGCTT
GhbHLH021-F	GGTCTTTTGCTCTCCATAATC
GhbHLH021-R	ATCATGTTGGGGAAGCCAGT
GhbHLH026-F	ACGTTGGATGGTACAGGTGGTGAA
GhbHLH026-R	CTGGAGACCCTTGCTTCCCTG
GhbHLH035-F	GAGTCACAGCAAAGCTGAGA
GhbHLH035-R	GCAGCAAATGACCTTACAAC
GhbHLH057-F	GCTTAATTCCTGGTGTAGCG
GhbHLH057-R	ACCACGACTGCGTAAACTTC
GhbHLH059-F	GAGGACTAACATCAGGAACA
GhbHLH059-R	CATCATGGGGACTGCTCATA
GhbHLH069-F	GCAATGGTTCAGGAAACATG
GhbHLH069-R	CGACTTGAAGTGTGCATGAA
GhbHLH083-F	GTATTCTCCCATGGCACTTG
GhbHLH083-R	GAATGTGACATGGACCTTAG
GhbHLH086-F	GCTGAGAGAAGGAGAGAAAG
GhbHLH086-R	CAGAAACTGCTGCAACCTTC
GhbHLH090-F	CAGTCCAGCGGCGGCGTTTC
GhbHLH090-R	CTCGGCGCTTTCGGCATCAA
GhbHLH093-F	GGATTTTAACATGGATAGTT
GhbHLH093-R	GGGCATAGACTGAGTGAAATA
GhbHLH096-F	CAGTGGAAGTGGGAAGCCC
GhbHLH096-R	TACCATAAGATATCCAGTCG
GhbHLH102-F	CACTATCATCAGTTGAGGAC
GhbHLH102-R	GTATCCGGTGGTTGCGAGTG
GhbHLH104-F	AACCTCCACCGCAAACGGA
GhbHLH104-R	GCTCTTAATTCAGCTGTAAAG
GhbHLH109-F	CTAGCAGAAGGTCGAGACAG

GhbHLH109-R	AACTGTGAGAGCCGTTCACT
GhbHLH113-F	GAGGACCTGAATCGACCTT
GhbHLH113-R	GCTGAACCACCATGTTGTTG
GhbHLH120-F	GATGAAACTTTTACAGGATC
GhbHLH120-R	TAGGTTGAGTATGCTAGAAC
GhbHLH132-F	GGGCTTCCTATGAACAATGT
GhbHLH132-R	ATGCTTTGGAGGTCATCAT
GhbHLH145-F	GATGCCACCATCTACAAGTAT
GhbHLH145-R	GCAAACCCATTGAAACAGCTA
GhbHLH147-F	CCTTGCACTAGCCGTGAAAGT
GhbHLH147-R	ATCATCCCTGCAGGTGCAATG
GhbHLH148-F	ATCATGTTGAAGCCGAGAGG
GhbHLH148-R	CCTGCCGAATCTCTCGAAGT
GhbHLH149-F	TGAGGAAGAGCAACAGGCAG
GhbHLH149-R	AGACGACATGCTTGCATGAC
GhbHLH150-F	CCAATCATATCATCGACGTA
GhbHLH150-R	CGGGGTATAATCAGATCAAG
GhbHLH151-F	GGAGAGCAGAGGCCTTTACA
GhbHLH151-R	CGAACATTTGGATCATATAG
GhbHLH156-F	GCATAGCAATATGTCTAATGGGAC
GhbHLH156-R	GTAATGCCATATCTTGGCTCTG
GhbHLH157-F	CCAAAGGGATTGCAGAAGCAA
GhbHLH157-R	GCTATCGCGAGGACCTTGAAT
GhbHLH174-F	GGAGCTAACCCGAACCCCTT
GhbHLH174-R	GTTGGTGGTGATGGGAAGTG
GhbHLH184-F	CACAATTTCAATCCAGCCCTA
GhbHLH184-R	AGCTGAGCCGGGTTGAACCA
GhbHLH188-F	GACTGATAGAATTGGAAACC
GhbHLH188-R	AGCCTGAGCTGCTCCTGTGT
GhbHLH195-F	CCCCAAGAAACTAGGGTTGT
GhbHLH195-R	AGGTGAGGCAGAACATGATC
GhbHLH200-F	GCTGTTGAGCGGAAGTTGAAGG
GhbHLH200-R	GACATTAGAGGGAAGGCGATC
GhbHLH202-F	CCTAGCTTCATGCCTCCTGCG
GhbHLH202-R	GGAGTACATGATCCTGTGAG
GhbHLH206-F	GAAAGCAGACAAGGAGCAAT
GhbHLH206-R	AACGTGATCCTGTGATGTAT
GhbHLH208-F	GCACAACCTAGCTTCATGCCG
GhbHLH208-R	GGAGGACATGGTCCTGTGAA
GhbHLH212-F	GCTCTGAGGATCTGGAACAG
GhbHLH212-R	ATATTTAACATGAATACCTC
GhbHLH217-F	GGGAGGATGATTTACAGAGC
GhbHLH217-R	TACAAATCAGGATTTTGTTG
GhbHLH229-F	AACTGTGGATTCTAACGAC

GhbHLH229-R	TCAATCATTTCGCCCATCT
GhbHLH241-F	GCTACAGAATGCGGATTCAAA
GhbHLH241-R	GGTCGTTAACGACGGATACGCTA
GhbHLH256-F	GCTAATGGAGGAAGACATG
GhbHLH256-R	TTGTAGCCGACTGGACAGT
GhbHLH259-F	AGATATGGGCGCGTTTTGCG
GhbHLH259-R	CACTGTAAGCTACTGGCTGT
GhbHLH261-F	GTGCCACAGTCTTTGGTTTT
GhbHLH261-R	CAATTGCTTAATTACTTCAC
GhbHLH264-F	CGTGATGAGAAGCAGAGGCT
GhbHLH264-R	GCGGAGCATATGATCCTGTG
GhbHLH268-F	GCAAGGAAGAGAAGAGAAAG
GhbHLH268-R	TTAACCAAGCTGCATCTTCG
GhbHLH281-F	CAGCAGTTGTCGGGTTCAAT
GhbHLH281-R	CAAAGCCCCGGTTTTAACCG
GhbHLH282-F	GAATGACGGCTTGCCCAGAG
GhbHLH282-R	TTGGCACAGCTAGAATCTGC
GhbHLH287-F	CTGATGCAGGGGATGTTGAGTT
GhbHLH287-R	GTGGAGCAGCTGCATACTGTC
GhbHLH288-F	CTGATGCAGGGGATGTTGAGTG
GhbHLH288-R	GAGCTTCAGTTGGACCTCCA
GhbHLH290-F	GGCAGTGATGGATCATCAGGAAAC
GhbHLH290-R	CCGACTTGAAGTGTACATGATATT
GhbHLH293-F	GGCTGCCGCCATCGGCACCG
GhbHLH293-R	AATGAACGGAACGGATTTAG
GhbHLH296-F	CTGGACTTTAACATGGATGG
GhbHLH296-R	AGGCATAGATGGAGTGAAATG
GhbHLH305-F	GGAAGAAGCCAACCAGCATG
GhbHLH305-R	GTGTGTGGTAAATGGCCTTG
GhbHLH306-F	GCTGTTGTGTGAGGAATGTAGT
GhbHLH306-R	CTAGCTTGTAGCCTTGGATTG
GhbHLH308-F	CAGTGGAAGTGGGAAGCCA
GhbHLH308-R	GATTGCAGAATTTGCACAAGCG
GhbHLH313-F	TCAGTAATATTCCCAAAGGG
GhbHLH313-R	AGTCTCCCCTTTACATGCCC
GhbHLH319-F	GAGTGCATCGATTCAGGTAAACC
GhbHLH319-R	ACCATTCGTGCGCGCCAAGC
GhbHLH320-F	AACCTCCACCGCAAACGGC
GhbHLH320-R	GCTCTTAATTCAGCTGTAAAC
GhbHLH336-F	CTTTGGGCCAATGGTGTGTT
GhbHLH336-R	GAATTTGATCATTCCTATGC
GhbHLH351-F	CCTGCAAAATCCGAGCCAAA
GhbHLH351-R	AATATGCTGCACAGCCAAA
GhbHLH352-F	GGGCTTCCTATGAACAATGG

GhbHLH352-R	ATGCTTTGGAGGTCATCAC
GhbHLH354-F	GTGAGGAGAAGCTTGTACTG
GhbHLH354-R	AGACGTATCCCGTGCTGATG
GhbHLH359-F	GCAGGTAGGAATACGAGTTT
GhbHLH359-R	TACAGAAATGGTTCGTGCAC
GhbHLH373-F	TCATGTTGAAGCTGAGAGGC
GhbHLH373-R	CCTGCCGAATCTCTCGAAGT
GhbHLH374-F	TGAGGAAGAGCAACAGGCAA
GhbHLH374-R	AGACGACATGCTTGCATGAT
GhbHLH375-F	CGACTTACATTCACCAGTCAAGC
GhbHLH375-R	CGCTTTCTTCGTTCTCTAGTG
GhbHLH377-F	AGAGAGCAGAGGCCTTTACA
GhbHLH377-R	CGAAAATTCGGATCAATAGT
GhbHLH378-F	GAAGCTAGGCCAAAGAAGAC
GhbHLH378-R	TGGCTCTCAACTCAGCTGCT
GhbHLH397-F	CGACTTACATTCACCAGTCAAAT
GhbHLH397-R	CGCTTTCTTCGTTCTCTAGTA
GhbHLH400-F	GATGCTACTGGTATGGCATT
GhbHLH400-R	TGCAATGGAGGCTCCATGCC
GhbHLH406-F	CGAAGTTAGACTGGCTACAG
GhbHLH406-R	GTTGTGATATTAGCATCAAT
GhbHLH409-F	AACAATTTCAATCCAGCCCTC
GhbHLH409-R	AGCTGAGCCGGGTTGAACCC
GhbHLH421-F	CGCCCAAGAAACCAGGGCTG
GhbHLH421-R	AGGTGAGACAGAACATGATC
GhbHLH428-F	CCTAGCTTCATGCCTCCTGCA
GhbHLH428-R	GGAGTACATGATCCTGTGAG
GhDWF4-F	GGCAGCAAAATAATGGAAGTCG
GhDWF4-R	TTTGAAGACTCTGATGGGTAGG
GhCPD-F	ATGCTCGCACGTTCAATCC
GhCPD-R	TCCGTTTCGACGCCGCACATTG
GhbHLH282-Up	GGATCCATGCAGCCTTGTAGTCGTGA
GhbHLH282-Dn	TCTAGATCACGGCTTGGAAACGGAAG
GhUBI1-F	CTGAATCTTCGCTTTCACGTTATC
GhUBI1-R	GGGATGCAAATCTTCGTGAAAAC
AtACTIN2-F	GAAATCACAGCACTTGCACC
AtACTIN2-R	AAGCCTTTGATCTTGAGAGC
